# Tunable expression rate control of a growth-decoupled T7 expression system by l-arabinose only

**DOI:** 10.1186/s12934-021-01512-7

**Published:** 2021-02-01

**Authors:** Patrick Stargardt, Gerald Striedner, Juergen Mairhofer

**Affiliations:** 1 enGenes Biotech GmbH, Mooslackengasse 17, 1190 Vienna, Austria; 2 Department of Biotechnology, University of Natural Resources and Life Sciences (BOKU), Muthgasse 18, 1190 Vienna, Austria

**Keywords:** *Escherichia coli*, enGenes-X-press, Growth-decoupled, *gp2*, BL21-AI, Recombinant protein production, Expression rate control, Resource reallocation, Flow cytometry, pBAD, l-Arabinose

## Abstract

**Background:**

Precise regulation of gene expression is of utmost importance for the production of complex membrane proteins (MP), enzymes or other proteins toxic to the host cell. In this article we show that genes under control of a normally Isopropyl β-d-1-thiogalactopyranoside (IPTG)-inducible P_T7-lacO_ promoter can be induced solely with l-arabinose in a newly constructed *Escherichia coli* expression host BL21-AI<*gp2*>, a strain based on the recently published approach of bacteriophage inspired growth-decoupled recombinant protein production.

**Results:**

Here, we show that BL21-AI<*gp2*> is able to precisely regulate protein production rates on a cellular level in an l-arabinose concentration-dependent manner and simultaneously allows for reallocation of metabolic resources due to l-arabinose induced growth decoupling by the phage derived inhibitor peptide Gp2. We have successfully characterized the system under relevant fed-batch like conditions in microscale cultivation (800 µL) and generated data proofing a relevant increase in specific yields for 6 different *Escherichia coli* derived MP-GFP fusion proteins by using online-GFP signals, FACS analysis, SDS-PAGE and western blotting.

**Conclusions:**

In all cases tested, BL21-AI<*gp2*> outperformed the parental strain BL21-AI, operated in growth-associated production mode. Specific MP-GFP fusion proteins yields have been improved up to 2.7-fold. Therefore, this approach allows for fine tuning of MP production or expression of multi-enzyme pathways where e.g. particular stoichiometries have to be met to optimize product flux.

## Background

Tunable control of gene expression for the overproduction of recombinant proteins in *Escherichia coli* (*E. coli*) is a promising strategy to further optimize soluble recombinant protein production (RPP) levels. This approach is of particular interest for membrane proteins (MP) or other difficult-to-express proteins (e.g. proteins rich in disulfide bonds) that tend to overwhelm host cell capacities [1]. This can be manifested by (i) overstraining the translocation capacities to the periplasm or the outer membrane [1–5], (ii) metabolic disturbances in the case that the protein of interest (POI) has enzyme characteristics [6], or (iii) competition for scarce amino acids or the overconsumption of particular amino acids or building blocks [7]. Based on these examples it becomes evident that for a particular POI there exists an optimal expression rate leading to maximal recombinant protein yield under given production conditions. In light of this, it is therefore important to use *E. coli* expression systems that provide tunability of gene expression. Although considered the gold-standard expression system in *E. coli* the widely used T7 expression system cannot be considered a tunable expression system per se, although widely used in such an approach. The T7 expression system exerts “all or none” inducibility that does not allow modulation of expression over a wide dynamic range. In recent years several systems for tunable gene expression or expression of toxic proteins have been developed.

For example, in the research of Miroux and Walker mutants of *E. coli* BL21(DE3) were isolated, which were generated through directed evolution approach, by expressing a particular MP in BL21(DE3) during ongoing cultivation in the presence of the inducer Isopropyl β-d-1-thiogalactopyranoside (IPTG) [8]. The so-named Walker strains C41 and C43, showed mutations in P_lacUV5_ which decreases expression of T7 RNAP and therefore reduces the transcription rate of the Gene of Interest (GOI). Recently published research by Kim et al. found further mutations responsible for a reduction in cellular toxicity caused by MP overexpression in C41/C43 strains, one occurring in *lacI* in the λDE3 chromosomal, which finally resulted in an even more reduced level of expressed T7 RNAP [9, 10].

Based on the observation made by the Walker strains, Wagner et al. developed the so-called Lemo21(DE3) strain, which works by the fact that the activity of T7 RNAP can be controlled by it natural inhibitor, T7 lysozyme, which was placed under the control of the titratable L-rhamnose inducible promoter (P_rhaBAD_) [11]. Lemo21(DE3) showed improved target protein yields, especially for MP.

The so-called Tuner(DE3) strain is a BL21(DE3) mutant that possesses a mutation in the lac permease (*lacY*), allowing for uniform uptake of the inducer IPTG, which results in a concentration-dependent, homogeneous level of induction [12].

Another approach to gain control of expression levels is by tuning the transcription rate of recombinant protein through repressor titration. Striedner et al. demonstrated that in fed-batch processes it is possible to control the expression level of a POI by feeding of the inducer IPTG at a constant ratio of IPTG to expected biomass and thereby generating a more stable and productive bioprocess [13].

A more recent approach is the RiboTite technology published by Morra et al.*,* which operates at the transcriptional and translational level by using standard IPTG inducible promoters and orthogonal riboswitches to generate a multi-layered modular genetic control circuit which allows for control of expression level of a POI [14].

As already mentioned, most MP are difficult-to-express in *E. coli* and the consequences of MP overexpression in *E. coli* have been reviewed already in detail [1, 15–17]. In this article, we focus on the characterization of a novel host strain which is capable of reallocation of metabolic resources by decoupling growth from recombinant protein production (RPP), as was shown recently with the enGenes-X-press technology [18], and additionally allow expression rate control in plasmid-based systems on the cellular level by titration of only one inducer (l-arabinose or IPTG). Briefly, by the use of the enGenes-X-press technology, host mRNA transcription can be inhibited by co-expression of a bacteriophage-derived *E. coli* RNA polymerase (RNAP) inhibitor peptide (Gp2), which binds the *E. coli* RNAP and therefore prevents σ-factor 70 mediated formation of transcriptional qualified open promoter complexes. Thereby, the transcription of σ-factor 70 driven host genes is inhibited, and metabolic resources can be exclusively utilized for the transcription of the GOI (by the orthogonal T7 RNAP) and translation into the final POI [18]. In the context of decoupling growth from product formation, several technologies have evolved in *E. coli* in recent years. For example, the so-called single-protein production system by Suzuki et al., which is based on BL21(DE3) strains encoding mazF, which leads to growth arrest by selectively degrading cellular 5′-ACA-3′-motif containing mRNAs [19]. Another example is the research by Izard et al., who designed an expression system that allows for growth arrest by external control of *rpoBC* genes, thereby controlling RNAP concentration and consequently bacterial growth of the system [20]. A more recent approach was shown by Li et al., who developed a CRISPR interference-based growth switches, allowing for knockdown of genes involved in the DNA replication or nucleotide synthesis machinery which are related to biomass growth of *E. coli* [21]. Nevertheless, as all presented systems can arrest cell growth, none of them is reported to be able to tune the expression rate on a cellular level and allow for growth decoupling simultaneously.

Furthermore, we wanted to investigate the possible promoter cross-talk of the two widely used sugar-based promoters, namely the l-arabinose inducible P_araBAD_ system and the conventional lactose controlled P_lacUV5_/P_T7-lacO_ promoter in expression systems, which use both systems simultaneously for control of T7 RNAP and GOI expression. It was already shown in previous studies that l-arabinose can induce lac-derived P_trc_ or P_lacUV5_/P_T7-lacO_ promoter in the *E. coli strains* JM109/JM109(DE3) and thereby allowing better soluble expression and less inclusion body formation of Penicillin G acylase (PAC) [22–24]. Further, it was shown that expression of PAC in *E. coli* strains MD∆P7 (mutation in *araC*) and MC4100 (mutation in *araD*) failed in showing equal PAC expression results from lac-derived P_trc_ with the induction of l-arabinose compared to JM109, hypothesizing that the derived metabolites of l-arabinose are responsible for the binding of the LacI repressor and that induction is not mediated by l-arabinose directly [23, 25]. In *E. coli* l-arabinose is converted into l-ribulose (araA), l-ribulose-5-phosphate (araB), and finally d-xylulose-5-phosphate (araD), subsequently drained into the pentose phosphate pathway.

Additionally, Narayanan et al. described a possible L-arabinose induction mechanism of lac-derived P_T7_ promoter in *E. coli* strain BL21-AI using T7 promoter-based vectors. The so-called restrained expression is able to control expression levels of the POI by the addition of l-arabinose to produce low levels of T7 RNAP and simultaneously skipping IPTG addition to profit from the occasional derepression on the lac operator site of P_T7_ to produce low levels of target mRNA [26].

## Results and discussion

As recently shown by our group in a growth decoupled expression system, induction of recombinant GFP expression from a pET-derived vector (expression is controlled by P_T7_) by the sole addition of l-arabinose, resulted in a comparable expression rate of soluble GFP but at the same time drastically reduced inclusion body formation, compared to induction with l-arabinose and IPTG or IPTG only. This growth decoupled expression system is based on *E. coli* strain BL21(DE3), which includes a chromosomal copy of T7 RNAP controlled by the IPTG inducible P_lacUV5_ and additional has a deletion of *araBAD* gene cluster to avoid metabolization of l-arabinose. Thereby, we can rule out that induction of lac-promoters is due to an ara*BAD*-derived l-arabinose degradation product (as suggested by Narayanan et al.) [23, 25]. Instead, it seems that l-arabinose can indeed bind or interact with LacI and thereby allows transcription of P_lacUV5_ controlled T7 RNAP, and in the same way derepressing the P_T7_ controlled GOI located on the pET-based plasmid. Although the exact mechanism remains elusive, this strain can substitute the inducer IPTG for l-arabinose. Interestingly, we have already shown a significant decrease in inclusion body formation with L-arabinose induction only compared to combined induction with l-arabinose and IPTG [18]. We believe that this might be due to a lower level response of the “sub-optimal” inducer l-arabinose compared to IPTG and/or LacI stability in this particular growth-decoupled strain.

### Genetic engineering of the host strain

**Fig. 1:**
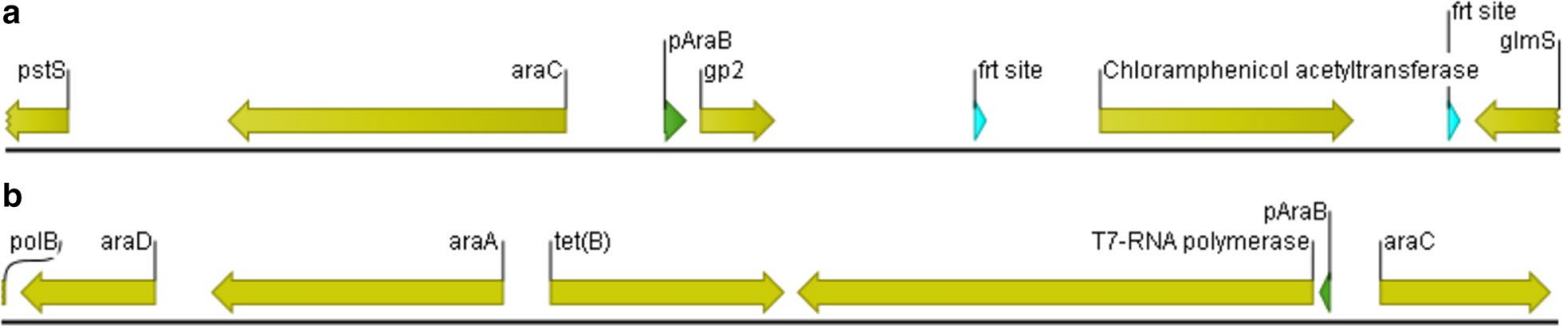
**a** Genetic modification introduced at the attTn7 site in between the genes *pstS* and *glmS* of *E. coli* strain BL21-AI<*gp2*>. **b** Genetic modification introduced at the araBADC site in the genome of *E. coli* strain BL21-AI

This finding led us to the idea of designing an expression strain that is based on the beforementioned concept. We, therefore, have chosen *E. coli* strain BL21-AI as a chassis strain. As shown in Fig. 1a, this strain already includes a chromosomal copy of the T7 RNAP, controlled by the l-arabinose inducible P_araBAD_ system, inserted at the *araB* site on the genome. By deletion of *araB,* the strain is no longer able to metabolize l-arabinose. Furthermore, to allow reallocation of host resources and growth-decoupled recombinant protein production, we inserted a gene copy of phage T7 derived inhibitor Gp2 [27–30] under control of P_araBAD_ promoter system at the attTn7 site (Fig. 1b) on the host chromosome by homologous recombination [18]. By that, we are able to induce growth arrest by expression of Gp2 and simultaneously control expression levels of recombinant proteins, where the transcription of a GOI is controlled by the P_T7_ promoter (located on conventional pET-derived vectors), by addition of different concentrations of l-arabinose only.

According to the recently published genome sequence of BL21-AI from Bhawsinghka et al., 7 point mutations (Additional file 1: Figure S1) were found in the T7 RNAP sequence, compared to BL21(DE3) [31]. This resulted in an amino acid exchange in the N-terminal domain of T7 RNAP, with the following residues affected: AA92(M → K) and AA246(P → L—both located in the promoter binding subdomain, as well as AA165(Y → N) and AA176(R → H)—both located in subdomain H, along with amino acid exchanges in the polymerase domain of T7 RNAP, with following residues affected: AA333(R → K), AA580(V → E) and AA717(G → E) [32, 33]. These mutations possibly have an impact on transcription activity by T7 RNAP and finally affect transcript levels of the GOI [34]. Nevertheless, as we directly compare the native BL21-AI strain and the developed strain BL21-AI<*gp2*>, this difference is of minor importance for our study (although it may impact improved MP production in BL21-AI compared to e.g. BL21(DE3)).

### Analysis of expression kinetics by omission of IPTG and usages of l-arabinose only for induction of lac-derived P_T7_ promoter system

**Fig. 2:**
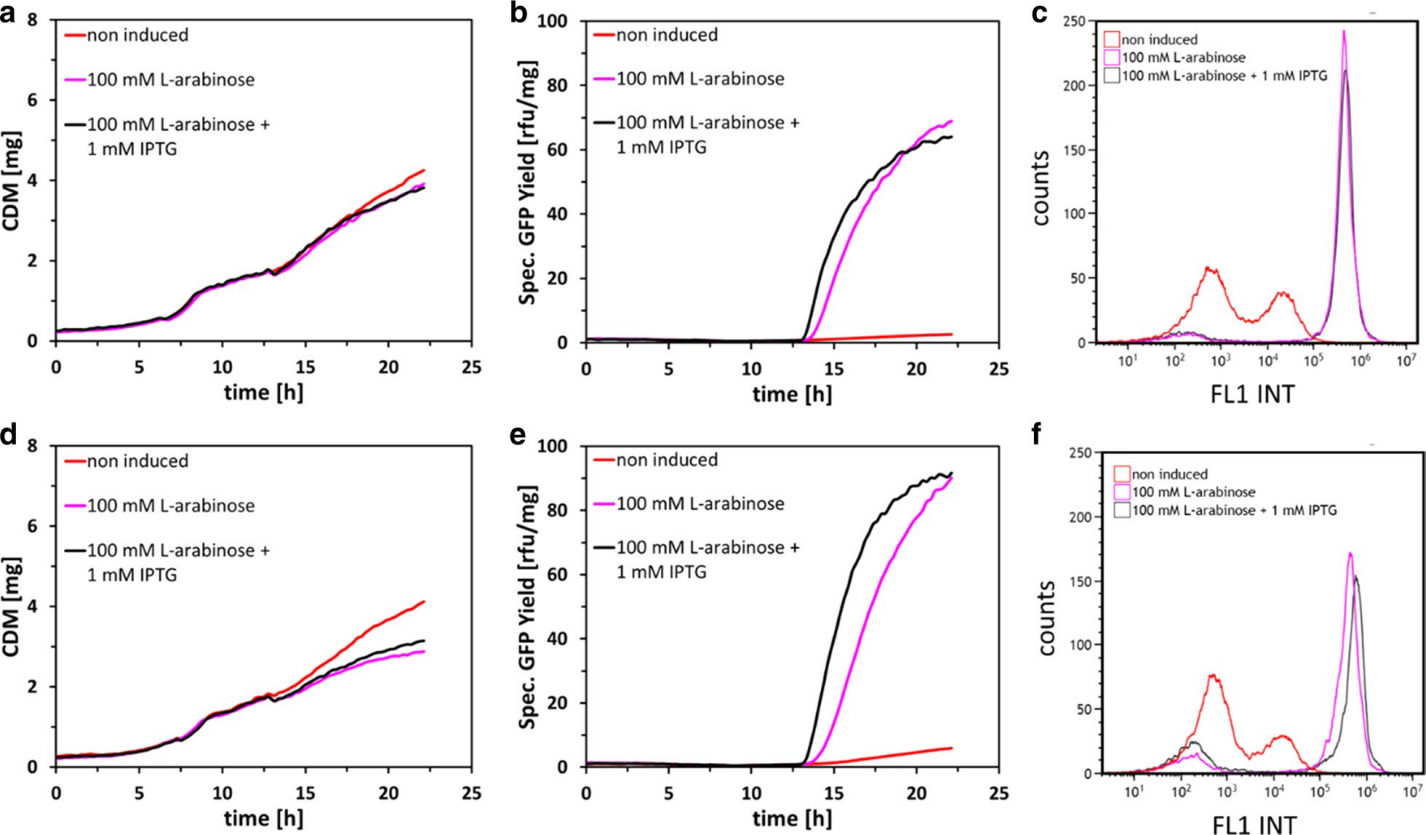
Process characteristic showing product formation kinetics and flow cytometry analysis of *E. coli* strains BL21-AI (**a–c**) and BL21-AI<*gp2*> (**d–f**) expressing GFPmut3.1 during fed-batch like cultivation. Induction was performed with 100 mM l-arabinose and 100 mM l-arabinose + 1 mM IPTG. The mean CDM [mg] and mean specific GFP yield [rfu/mg] represents duplicate samples, error bars omitted as standard error of mean was below 4%. Flow cytometry experiments were performed in duplicate, results from a single experiment are presented

In the next step, we analyzed the effect of IPTG omission on growth and product formation kinetics on *E. coli* strains BL21-AI and BL21-AI<*gp2*>, harboring the reporter plasmid pET30a(GFPmut3.1)cer. Therefore, we performed fed-batch-like cultivations in 48-well microtiter plates where we compared standard pulse induction (100 mM l-arabinose + 1 mM IPTG) with pulse induction of l-arabinose only (100 mM l-arabinose). As expected under fully induced conditions (Fig. 2a), growth kinetics of BL21-AI only slightly differs from non-induced conditions, whereas BL21-AI<*gp2*>showed decreased growth upon induction of L-arabinose (and IPTG) (Fig. 2d). Induction with 100 mM l-arabinose shows a delayed increase in specific GFP yields in BL21-AI and BL21-AI<*gp2*>compared to combined induction with 100 mM l-arabinose and 1 mM IPTG. Nevertheless, comparing the two different induction conditions on productivity (specific GFP yields), both conditions reached nearly the same values at end of the process, with BL21-AI yielding 68.9 rfu/mg (100 mM l-arabinose) and 64.0 rfu/mg (100 mM l-arabinose + 1 mM IPTG) (Fig. 2b), respectively BL21-AI<*gp2*>which yielded 90.4 rfu/mg (100 mM l-arabinose) and 91.7 rfu/mg (100 mM l-arabinose + 1 mM IPTG) (Fig. 2e), showing 33.1% higher specific GFP expression compared to BL-21-AI. Additionally, flow cytometric analysis was performed to check if applied induction strategies led to population heterogeneities or a different induction behavior (Fig. 2c, f). After 10 h of recombinant GFP expression, induction with or without 1 mM IPTG showed no significant impact on the population distribution of BL21-AI and BL21-AI<*gp2*>, demonstrating that induction with l-arabinose can serve as a substitution to combined induction with 1 mM IPTG.

Furthermore, we observed a higher level of basal expression of BL21-AI (Fig. 2b, c, non-induced) and BL21-AI<*gp2*> (Fig. 2e, f, non-induced) when compared to the conventional BL21(DE3)/pET (Fig. 4b, non-induced) expression system under fed-batch like cultivation conditions. This is in stark contrast to the previously reported low level of basal expression that has been observed in classical batch cultivations [35, 36]. This is due to the regulative nature of the positively controlled l-arabinose operon [37, 38], as it contains a functional CAP site, otherwise deleted within the P_lacUV5_ version found in the DE3 expression systems [4, 39]. Unlike P_lac_, P_lacUV5_ works independently of activator proteins or other cis-regulatory elements and has lost responsiveness to catabolite repression. The result is less sensitivity to increased cAMP levels that occur upon transition from the non-glucose-limited state (Batch) to the glucose-limited state (Fed-batch), and consequently lower basal level expression, compared to P_araBAD_, upon entering glucose-limited conditions [40–42].

### Tuning recombinant protein expression on population level

For P_araBAD_ vector-based expression system, the phenomenon of all-or-none induction, which leads to great heterogeneity in cellular populations upon titration of inducer, is well described [35, 43–45]. In this context, several approaches have been developed within recent years to overcome this specific problem, either by decoupling the expression of genes encoding l-arabinose transporters and metabolic proteins by placing the genes on a second vector [44] or by supplementing l-arabinose/d-glucose feeding media during fed-batch processes to tune expression level via the specific substrate uptake rate of the inducer [46]. However, both options have their downsides. Option one creates an additional metabolic burden due to constant overexpression of transporter genes and option two needs to feed expensive l-arabinose as a growth media supplement.

Based on the finding that 100 mM l-arabinose can serve as a substituent for IPTG to obtain fully induced conditions, we wanted to evaluate if we can fine-tune expression levels by different l-arabinose concentration pulses. Therefore, we performed again fed-batch-like cultivations in 48-well microtiter plates with *E. coli* strains BL21(DE3), BL21-AI, and BL21-AI<*gp2*>, harboring the reporter plasmid pET30a(GFPmut3.1)cer. At the end of batch phase, we induced the strains with 0.025 mM (0.000375% w/v), 0.25 mM (0.00375% w/v), 1 mM (0.015% w/v), 2.5 mM (0.04% w/v), 5 mM (0.08% w/v) and 100 mM l-arabinose (1.5% w/v). The production phase lasted for approximately 10 h.

**Fig. 3:**
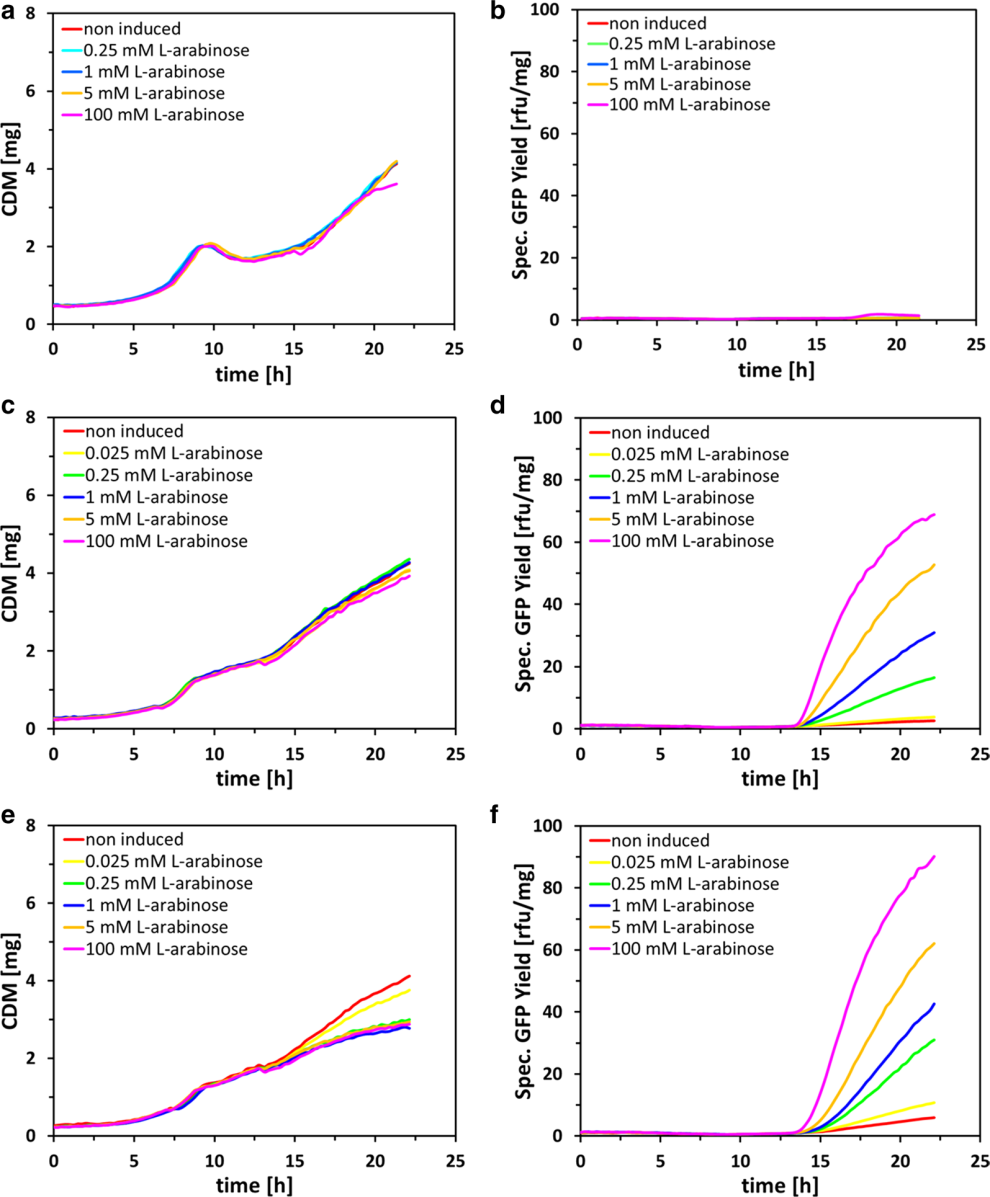
Process characteristic and product formation kinetics of *E. coli* strains BL21(DE3) (**a, b**), BL21-AI (**c, d**) and BL21-AI<*gp2*> (**e, f**) expressing GFPmut3.1 during fed-batch like cultivation. Induction was performed with different l-arabinose concentrations (0.025, 0.25, 1, 5, 100 mM). The mean CDM [mg] and mean specific GFP yield [rfu/mg] represents duplicate samples, error bars omitted as standard error of mean was below 4%

For strain BL21(DE3) induction with 100 mM L-arabinose only showed a slight increase of specific GFP fluorescence (1.4 rfu/mg) (Fig. 3b), which was to be expected as the genotype of BL21(DE3) does not contain any mutations of the l-arabinose operon genes (*araBAD*) which could avoid l-arabinose metabolization. For strains BL21-AI and BL21-AI<*gp2*>, which contain a deletion of the *araB* gene, no such effect could be observed. Furthermore, both strains showed responsiveness to inducer titration, yielding specific GFP expression in a range of 3.9–68.9 rfu/mg for BL21-AI (Fig. 3d), respectively 10.8–90.2 rfu/mg for BL21-AI<*gp2*> (Fig. 3f) with 0.025–100 mM L-arabinose. We have thereby proved that specific GFP expression can be controlled on the whole population level by varying concentrations of L-arabinose. As mentioned above, BL21-AI<*gp2*> (90.2 rfu/mg) showed higher specific GFP expression compared to BL21-AI (68.9 rfu/mg) with 100 mM L-arabinose. This trend could also be observed for lower l-arabinose concentration.

With each concentration tested, specific GFP expression was higher in BL21-AI<*gp2*>compared to the BL21-AI reference experiment. This observation can be explained by the capability of this technology to allow inducible resource reallocation by decoupling growth from RPP. This circumstance can also be seen in the course of CDM after induction with l-arabinose, where only BL21-AI<*gp2*> showed a reduction in CDM accumulation (Fig. 3e) compared to BL21-AI (Fig. 3c) or BL21(DE3) (Fig. 3a), and simultaneously increased specific GFP yields.

**Fig. 4:**
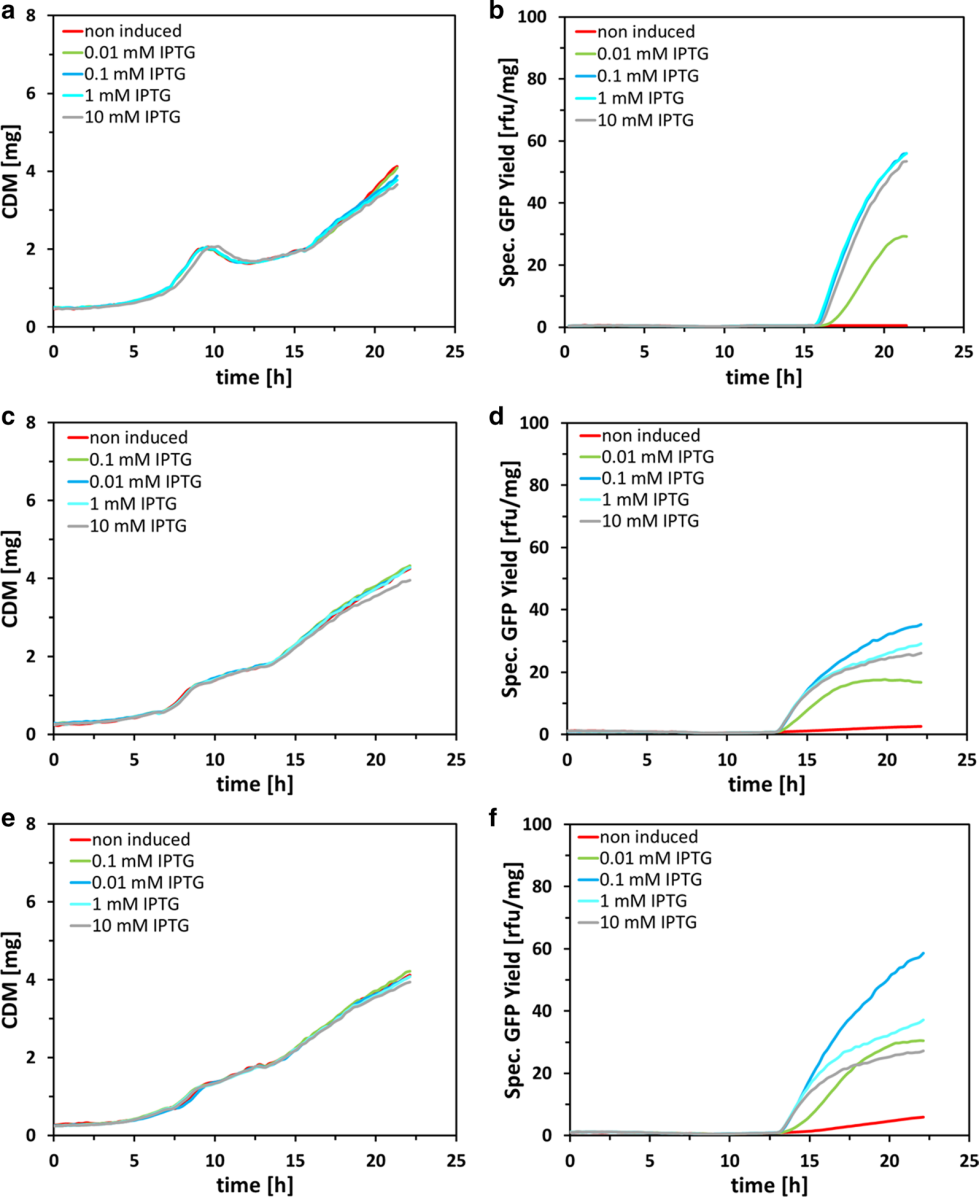
Process characteristic and product formation kinetics of E. coli strains BL21(DE3) (**a**, **b**), BL21-AI (**c**, **d**) and BL21-AI<*gp2*> (**e**,** f**) expressing GFPmut3.1 during fed-batch like cultivation. Induction was performed with different IPTG concentrations (0.01, 0.1, 1, 10 mM). The mean CDM [mg] and mean specific GFP yield [rfu/mg] represents duplicate samples, error bars omitted as standard error was below 4%

In the next step, we analyzed the impact of the basal expression level of the P_araBAD_ controlled T7 RNAP in BL21-AI and Bl21-AI<*gp2*> on growth and product formation kinetics. By adding different IPTG concentrations (10 mM–0.01 mM) and direct comparison to BL21(DE3) under the same induction and cultivation conditions, we wanted to gain insight into the tunability of GFP expression on the population level by omitting the inducer l-arabinose. In the case of BL21(DE3), tuning of expression level was impossible as only the addition of 0.01 mM IPTG resulted in a lower specific GFP yield (29.2 rfu/mg) compared to induction with 0.1–10 mM IPTG, which resulted in comparable high specific GFP yields of 56–53.1 rfu/mg (Fig. 4b). Interestingly, BL21-AI showed improved tunability of GFP expression compared to BL21(DE3) but in more or less reversed order regarding IPTG concentration. As shown in Fig. 4d, highest specific GFP expression was observed with 0.1 mM IPTG (35.3 rfu/mg), followed by 1 mM (29.2 rfu/mg), 10 mM IPTG (26.1 rfu/mg) and 0.01 mM IPTG (16.8 rfu/mg). The possible explanation for this phenomenon is that IPTG can induce gene expression of GOI, located on a pET-based plasmid, by derepressing P_T7_, through binding to LacI, but simultaneously IPTG is acting as an inhibitor of the P_araBAD_ system, which controls the expression of T7 RNAP [47]. According to Schleif et al. it is not surprising that IPTG can bind AraC as IPTG possesses a D‐galactose moiety, and the ring structures of L‐arabinose and D‐galactose are showing high similarity [38]. Therefore, with increasing concentration of IPTG we get a decreased amount of T7 RNAP followed by less mRNA of the GOI and conclusively decreasing specific GFP yield. Similar results could be observed for BL21-AI<*gp2*>, 0.1 mM IPTG resulted in the highest specific GFP concentration with 58.6 rfu/mg followed by 1 mM (37.2 rfu/mg), 0.01 mM (30.5 rfu/mg) and 10 mM IPTG (27.3 rfu/mg) (Fig. 4f).

The in general higher specific GFP yield with the induction of IPTG, compared to BL21-AI, seems to be the results of increased basal level expression of T7 RNAP (Fig. 4d, f). Basal level expression was lowest in BL21(DE3) with a specific GFP yield of 0.2 rfu/mg (Fig. 4b), followed by strain BL21-AI (2.6 rfu/mg) (Fig. 4d) and BL21-AI<*gp2*> (5.9 rfu/mg) (Fig. 4f), showing that the P_araBAD_ system exhibits higher derepression than the P_lacUV5_ system, in glucose-limited fed-batch processes.

Only minor influence on growth kinetics was observed in all three strains (Fig. 4a, c, and e), with the biggest impact caused by induction with 10 mM IPTG. This can be explained by the possible toxicity of IPTG to the growth of *E. coli,* which is has been repeatedly reported [48–54].

### Tuning recombinant protein expression on cellular level—flow cytometric analysis

By adapting different l-arabinose or IPTG concentrations, it is possible to fine-tune protein production in BL21-AI and BL21-AI<*gp2*> at the population level. But more importantly, is the ability to control transcription rate on a cellular level. To maintain a robust and reliable bioprocess, it is essential to understand cellular behavior on the population level by titration of inducer concentrations. Therefore, we performed flow cytometric analysis on the population distribution of *E. coli* strains BL21(DE3), BL21-AI, and BL21-AI<*gp2*>, affected by different concentrations of l-arabinose or IPTG, at the end of glucose-limited fed-batch cultivation.

**Fig. 5:**
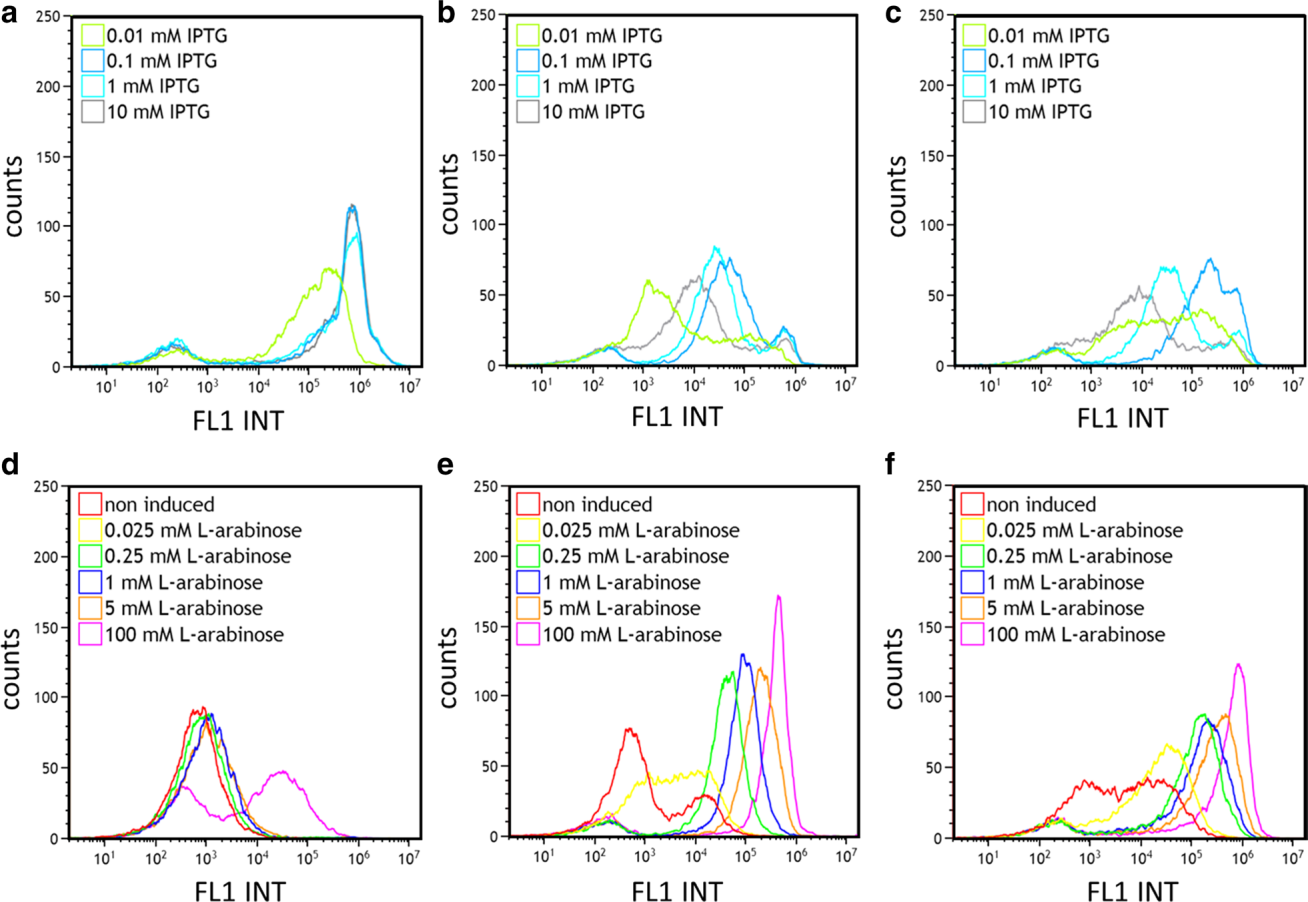
Flow cytometry analysis of single‑cell expression of GFPmut3.1 by Strain BL21(DE3) (**a**, **d**), BL21-AI (**b**, **e**) and BL21-AI<*gp2*> (**c**, **f**) during fed-batch like cultivation. Induction was performed either with IPTG (0.01, 0.1, 1, 10 mM) only (**a**–**c**) or l-arabinose (0.025, 0.25, 1, 5, 100 mM) only (**d**–**f**). Experiments were performed in duplicate. Results from a single experiment are presented

As already shown on the population level, induction of BL21(DE3) with different IPTG concentrations did not result in different GFP expression levels but triggered population inhomogeneities (Fig. 5a). According to Schuller et al., further dilution of IPTG will lead to all-or-none induction behavior, which results in a mixture of fully, partially and non-induced cells, especially at very low IPTG concentrations (0.005 mM) [55]. Compared to strains BL21-AI and BL21-AI<*gp2*>, induction with different IPTG concentrations resulted in a tunability of GFP expression, but at the same time exhibiting all or none induction on the cellular level as at least two distinct sub-populations were visibly at any given IPTG concentration (Fig. 5b, c). Furthermore, flow cytometry analysis proved that higher IPTG concentration resulted in lower GFP expression in BL21-AI and BL21-AI<*gp2*>, which was already observed on the population level. Therefore, we conclude, that IPTG can indeed inhibit basal expression of P_araBAD_ controlled T7 RNAP and by that decrease GFP expression. Those results confirmed that even with a basal level expression of T7 RNP, expression rate control on the cellular level is not possible, as already reported by several authors [36, 56–61].

Induction of BL21(DE3) with l-arabinose concentrations below 100 mM showed no expression compared to non-induced control (red graph) (Fig. 5d), which can be explained due to the ability of BL21(DE3) to metabolize the inducer l-arabinose. Furthermore, induction with 100 mM l-arabinose forced the formation of two distinct sub-populations of which one showed no increase in fluorescence intensity and one with a minor increase, especially compared to the result from fully induced conditions with IPTG (≥ 1 mM). Those results showed that l-arabinose can be used as an IPTG-substitute to induce transcription of P_lacUV5_ controlled T7 RNAP and to derepress P_T7_ controlled GOI, but in the same time exhibiting problems as strains with intact l-arabinose operon showed all-or-none induction behavior.

As the genotype of BL21-AI and BL21-AI<*gp2*> shows deletion of *araB* and insertion of T7 RNAP under control of P_araBAD_ system, induction with l-arabinose showed a significant step-up in the ability to fine-tune expression rate by varying inducer concentration (0.025–100 mM), compared to BL21(DE3). BL21-AI was able to maintain single population distribution during GFP expression with concentrations of ≥ 0.25 mM l-arabinose. Particularly induction with very low inducer concentration (0.025 mM l-arabinose) (yellow graph) showed a broadening of the population, which indicates a possible mixture of partially induced cells (Fig. 5e). Nevertheless, in the range of 0.25–100 mM l-arabinose BL21-AI was able to maintain single population homogeneity and shift fluorescence intensity according to l-arabinose concentration.

**Fig. 6:**
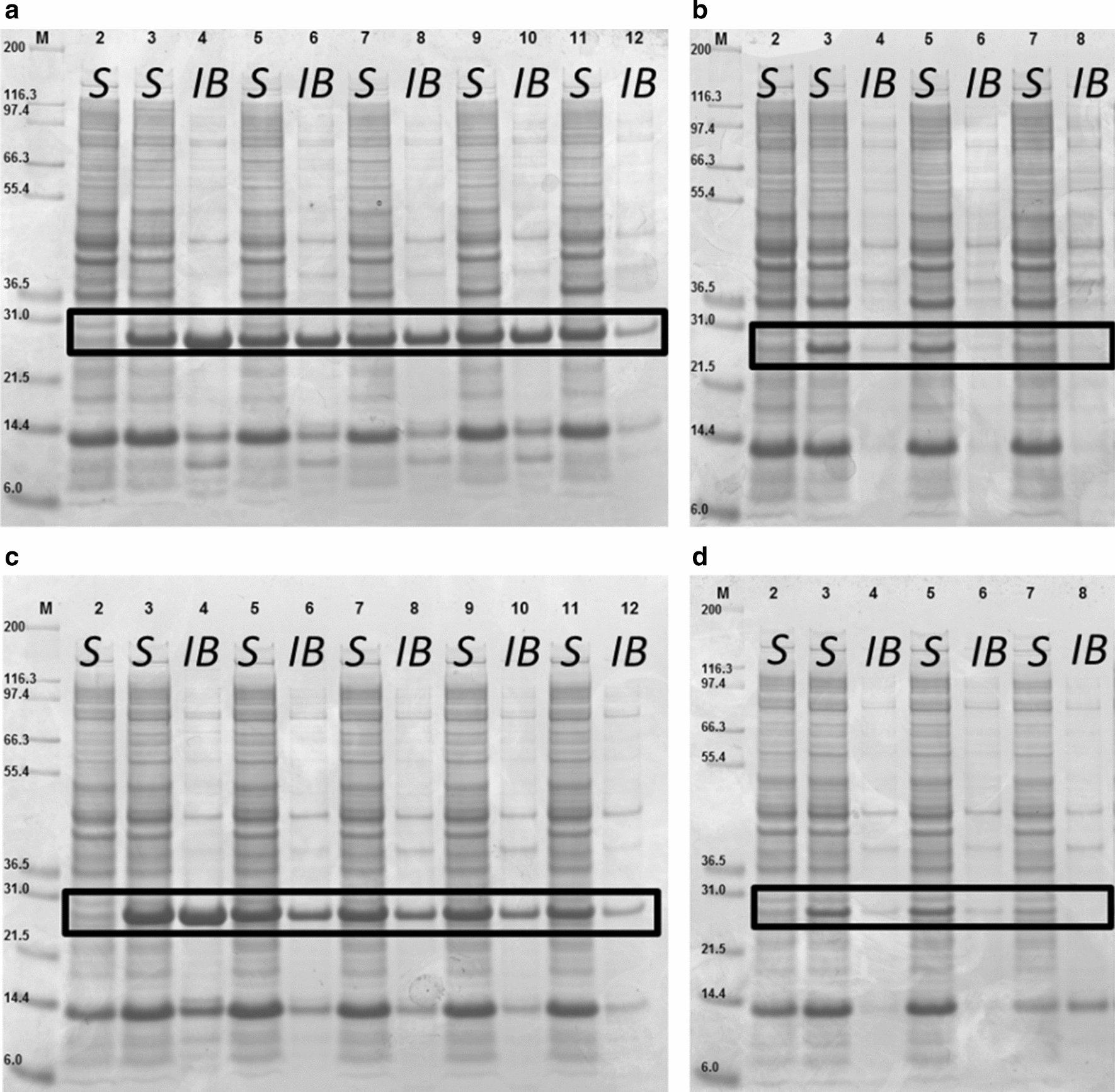
SDS-Page analysis of end samples (1 mg) from fed-batch like cultivation of E. coli strain BL21-AI (**a**, **b**) and BL21-AI<*gp2*> (**c**, **d**) expressing GFPmut3.1, showing distribution of soluble (S) and insoluble (IB) GFP**. **Lane M, molecular weight marker (NovexMark12 Unstained Standard); Induction was performed with (**a**, **c**) Lane 2: non-induced; Lane 3/4: 100 mM l-arabinose + 1 mM IPTG; Lane 5/6: 100 mM l-arabinose; Lane 7/8: 50 mM l-arabinose; Lane 9/10: 25 mM l-arabinose; Lane 11/12: 5 mM l-arabinose; (**b**,** d**) Lane 2: non-induced; Lane 3/4: 1 mM L-arabinose; Lane 5/6: 0.25 mM l-arabinose; Lane 7/8: 0.025 mM L-arabinose

Expression of GFP in growth decoupled strain BL21-AI<*gp2*> resulted in a homogeneous population at any given l-arabinose concentration (0.025–100 mM) (Fig. 5f), indicating that it is possible to maintain higher population homogeneity with this strain compared to BL21-AI, probably due to cessation of cell growth upon induction of the RNA polymerase inhibitor peptide Gp2. Furthermore, it was shown (Fig. 3d, f) that BL21-AI<*gp2*> was able to increase specific GFP yields during growth decoupled production at the same induction conditions. This was shown in flow cytometric analysis as well, as BL21-AI<*gp2*> population is yielding higher fluorescence intensity than BL21-AI. Additionally, SDS-Page analysis of strain BL21-AI (Fig. 6a, b) and BL21-AI<*gp2*> (Fig. 6c, d) revealed that by reducing expression rate, the formation of inclusion bodies is drastically reduced or even avoided, proving that tuning of transcription level of the GOI can help to avoid overburden of the cellular resources of the host.

**Fig. 7:**
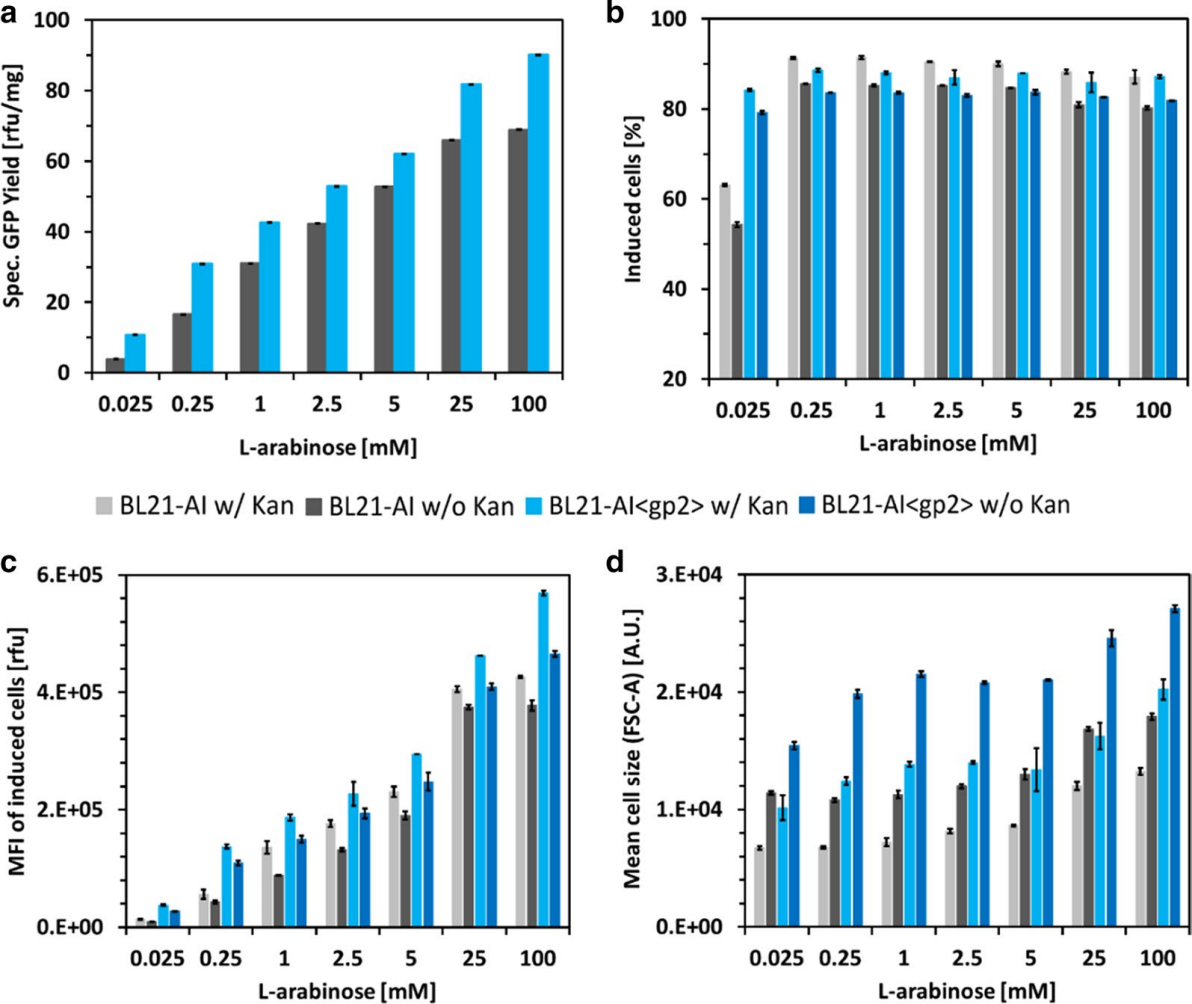
**a **Specific GFP expression as function of different inducer (l-arabinose) concentrations from fed-batch like cultivation of *E. coli* strain BL21-AI and BL21-AI<*gp2*> harboring the pET30a(GFPmut3.1)cer plasmid. **b** Percentage of induced cells from flow cytometry analysis in cultivations with (w/) or without (w/o) addition of 50 µg/mL kanamycin to media. **c** Flow cytometry analysis of MFI of induced cell population. **d ** Flow cytometry analysis of cell size shown as geometric mean of FSC channel. Experiments were performed in duplicate. Error bars indicate standard error of the mean (n  = 2)

In general, induction of GFP with l-arabinose resulted in a shift of the population to higher fluorescence intensities. Still, at any given l-arabinose concentration, a small subpopulation of non-induced cells was observable (Additional file 1: Figures S2, S3, S4, S5, S6, S7, S8, S9, S10). The fraction of non-induced cells was dependent on the strain and cultivation condition (with or without kanamycin addition to media) (Fig. 7b). For cultivations without the addition of kanamycin [KanR located on pET30a(GFPmut3.1)cer] to media, the fraction of non-induced cells was determined as ∼ 21% for BL21-AI and ∼ 18% for BL21-AI<*gp2*>. The addition of 50 µg/mL kanamycin to the media resulted in a decrease of non-induced cells to ∼ 14% for BL21-AI and ∼ 12% for BL21-AI<*gp2*>. Sagmeister et al. showed similar findings during GFP expression (from P_araBAD_ vectors system) in mixed-feed (l-arabinose/glucose) fed-batch cultivations of *E. coli* strain C41 and described the non-induced population as “nascent” cells [46]. We believe that this population consists of non-plasmid-bearing cells, as the addition of kanamycin can reduce the percentage of non-induced cells to a minimum but not avoid it, which could be explained by the emergence of spontaneous kanamycin-resistant cells [62, 63]. Still, in all cultivations, the fraction showed a stable percentage of non-induced cells for any given l-arabinose concentration with the exception to strain BL21-AI and induction with 0.025 mM l-arabinose, where an increase in the population of non-induced cells to 46% (w/o kanamycin) and 37% (w/ kanamycin) was observed. BL21-AI<*gp2*> was able to control population homogeneity over a wider range of expression, which may be due to the inability of cell division upon induced growth arrest, compared to BL21-AI. Nevertheless, with the presented finding of the ability to induce lac-derived promoter systems by addition of l-arabinose, protein expression in both strains can be controlled on a cellular level by simple pulse induction of different amounts of l-arabinose. We have shown this on the level of GFP expression normalized to CDM (Fig. 7a) as well as using flow cytometry analysis of the mean fluorescence intensity (MFI) of the induced cell population of cells (Fig. 7c). Interestingly, differences in cell size between strain BL21-AI and BL21-AI <*gp2*> were observed by flow cytometry analysis (using forward scatter channel, FSC-A, Fig. 7d). Figur**e** 7d indicates a strain independent increase in cell size of approximately 56–75% compared to cultivations including kanamycin in the growth medium. Furthermore, upon expression of Gp2, strain BL21-AI<*gp2*> showed an increase in cell size by 116–153% compared to strain BL21-AI. Another parameter that affected cell size was l-arabinose concentration. Cultivation of strain BL21-AI induced with different concentrations of l-arabinose (0.025–100 mM) showed an increase in cell size by 0–57%. As the biggest increase in cell size for strain BL21-AI was observed upon induction with 5–100 mM l-arabinose, we speculate that inclusion body formation (Fig. 6a, b) might be the reason for this increase in cell size as comparable results have been reported in the literature [64]. Similar results were observed for strain BL21-AI<*gp2*> (cultivation without kanamycin, Fig. 7d), where the first increase in cell size was indicated upon induction with 0.025 mM l-arabinose and a second increase upon induction with 25 mM l-arabinose. The first increase in cell size is possible due to Gp2 overexpression and consequent elongation of cells (due to stalled cell division) [65]. The second increase in cell size correlates with the increase in inclusion body formation upon induction with 25 mM or 100 mM l-arabinose (Fig. 6c, d).

Flow cytometry analysis proved also increased levels of basal GFP expression by non-induced (red graph) BL21-AI (Fig. 5e) and BL21-AI<*gp2*> (Fig. 5f) cells in glucose-limited fed-batch processes, as both strains either showed distinct subpopulations of producing and non-producing cells (BL21-AI) or a mixture of partly induced cells (BL21-AI<*gp2*>), especially compared to non-induced (red graph) BL21(DE3) (Fig. 5d) cells, which showed no induction. For the production of toxic proteins in glucose-limited fed-batch processes, the usage of the P_araBAD_ system is not optimal, as already low basal expression levels of recombinant protein could hamper the growth of cells. This gives rise to a population of non-producing cells with the potential to displace slow growing producing cells. For BL21-AI<*gp2*> this issue of basal expression is solved by the capability of the system to shut-down cell division. Therefore, in combination with fine-tuning of expression levels of the GOI on a cellular level using different pulses of l-arabinose, this growth-decoupled approach allows for improving the production of otherwise difficult-to-express proteins.

### Growth decoupled production induced by different l-arabinose concentrations can drastically increase the production of membrane proteins

Overproduction of MP is often tricky as proteins must be expressed and inserted into the membrane to allow for production in a correctly folded state. Furthermore, screening for high-level MP expression can be cumbersome, as gel-based assays are often not very accurate, and fractioning of soluble, insoluble, and membrane fraction can be laborious and time-consuming [2].

Additionally, overproduction of MP in classical systems like BL21(DE3), where the T7 RNAP is controlled by the strong P_lacUV5_ system, can result in too high transcription levels of the GOI and by that leading to an overload of the translocation capacity which finally inhibits the production of heterologous MP in such organisms [2–4]. Improved *E. coli* production systems, which enable for decreased expression of T7 RNAP (e.g. C41(DE3), C43(DE3), and Lemo21(DE3)) address these particular problems [8, 11].

As tuning of transcript level and reallocations of resources seems to be the key to successful overexpression of MP, we decided to benchmark our developed strain BL21-AI<*gp2*> against BL21-AI, by expression of 6 different *E. coli* derived MP containing C-terminal GFP-fusions [66]. As was shown by Drew et al., GFP is an attractive indicator to monitor overexpression of correctly folded MP by measuring whole-cell fluorescence on microtiter plate dish format, as the GFP moiety only folds properly if MP is inserted into the membrane [3, 10, 67–69]. By this approach, we can directly follow the expression level of the different MP (Yhdy-GFP, Psta-GFP, Ylif-GFP, YdiK-GFP, Yhhj-GFP, and YfbF-GFP) during fed-batch-like cultivations in 48-well microtiter plates.

**Fig. 8:**
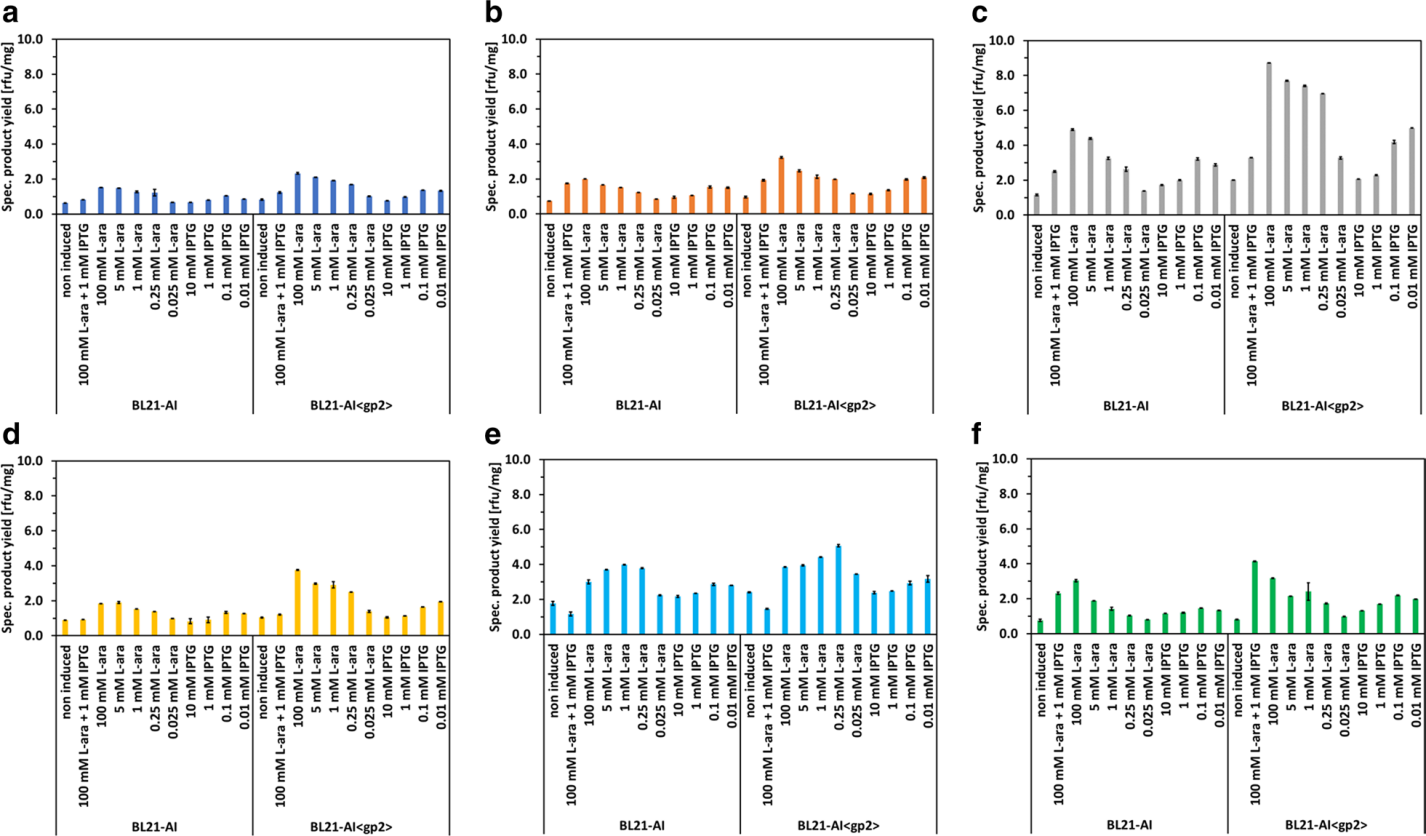
Comparison of specific product yields at the end of process of YhdY-GFP (**a**), PstA-GFP (**b**), YliF-GFP (**c**), YdiK-GFP (**d**), YhhJ-GFP (**e**) and YfbF-GFP (**f**) expressed during fed-batch like cultivation in *E. coli* strain BL21-AI and BL21-AI<*gp2*>). The mean specific product yield [rfu/mg] represents duplicate samples, error bars indicate standard error of the mean (n  = 2)

Specific product yields for all targets tested were significantly improved after 10 h of production, and *E. coli* strain BL21-AI<*gp2*> always outperformed parent *E. coli* strain BL21-AI (Fig. 8). Induction with 100 mM l-arabinose yielded increased specific product titers, compared to the standard induction scheme (100 mM l-arabinose + 1 mM IPTG), pointing out that a decrease in the transcription level of GOI increases final yields of the POI. Furthermore, flow cytometry analysis revealed that induction with 100 mM l-arabinose never showed the emergence of subpopulations, compared to induction with 100 mM l-arabinose + 1 mM IPTG, where distinct subpopulations were visible for expression of Yhdy-GFP (Additional file 1: Figure S12f) and YhhJ-GFP (Additional file 1: Figure S20c and f). Although, as already seen during expression of GFPmut3.1, BL21-AI<*gp2*> and parent strain BL21-AI showed distinct subpopulation in the non-induced state after 24 h of cultivation (Additional file 1: Figure S11c, f, i and l, S12, S13, S14, S15, S16, S17, S18, S19, S20, S21, S22c, f, i and l), which prove once more that expression system based on P_araBAD_ can exhibit a significant increase of basal level expression during glucose-limited growing conditions (e.g. fed-batch cultivation). Besides expression of YhhJ-GFP, a possible inner MP with several transmembrane domains [66, 70, 71], growth kinetics were not affected by basal level expression of POI, indicating that low-level expression of these POI is not toxic to the host. In both strains, BL21-AI and BL21-AI<*gp2*>, expression of YhhJ-GFP resulted in a significant growth decline after 20 h of cultivation (Additional file 1: Figures S19, S20).

With exception to YfbF-GFP, both strains showed the highest specific expression level for every POI with the induction of 100 mM l-arabinose, which imply that a decrease in transcript level is beneficial (compared to induction with 100 mM l-arabinose + 1 mM IPTG), but a further decrease in transcript level due to inducer titration is not necessary for an increase in specific productivity. YfbF-GFP, a possible inner MP with two transmembrane domains [66], showed the highest expression in both strains with 100 mM l-arabinose + 1 mM IPTG, indicating that overproduction of this specific protein is not limited by too high transcript levels of the GOI (Fig. 8).

Induction performed without l-arabinose showed similar behavior as already shown for the production of GFPmut3.1. The increase in specific protein yield by a decrease of IPTG concentration (10–0.01 mM) was also observed. For every tested POI, induction with 10 mM IPTG resulted in the lowest specific product concentrations (compared to other IPTG concentrations). Induction with 0.1/0.01 mM IPTG yielded the highest specific product concentration for every POI expressed in strain BL21-AI<*gp2*> and parent strain BL21-AI (Fig. 8). Nevertheless, induction approaches without l-arabinose showed an increase in population heterogeneity (Additional file 1: Figures S11, S13, S14, S15, S16, S17, S19, S20, S21, S22) compared to induction with l-arabinose, which again proves the superiority of this induction method, especially in combination with the possibility to reallocated host resources through induced growth arrest.

Depending on the induction condition, strain BL21-AI<*gp2*> showed a constant improvement of product yields and was able to increase specific product concentration for YhdY-GFP up to 1.5-fold, PstA-GFP up to 1.6-fold, YliF-GFP up to 2.7-fold, YdiK-GFP up to twofold, YhhJ-GFP up to 1.5-fold and YfbF-GFP up to 1.8-fold. Furthermore, specific product rates (data not shown) from performed fed-batch cultivation indicates that the production phase of strain BL21-AI<*gp2*> was still on-going upon termination of the process after 10 h of production, indicating that with increased production time, a further boost in productivity would be possible with our technology (Additional file 1: Figures S11, S12, S13, S14, S15, S16, S17, S18, S19, S20, S21, S22).

This data set confirms that growth decoupled MP production in combination with tunable expression of the GOI allows for significantly increased product yields. Reasons for this are manifold. For example, growth decoupled production frees up metabolic resources and makes MP specific chaperons available [72]. Other factors that explain the increase in productivity are a possible change in lipid composition due to the inability of growth decoupled cells to divide or the desaturation of the MP biogenesis machinery due to the inhibition of cellular MP expression [73, 74]. Finally, overexpression of MP induces stress which negatively affects the robustness of the production host and giving rise to non-producing, plasmid-free cells. Decoupling growth from MP production significantly lowers the likelihood of the development of a non-producing subpopulation of cells and therefore allows for higher specific protein yields [1, 11].

## Conclusion

In this paper, we show that by the construction of a new expression host BL21-AI<*gp2*>, based on the recently published approach of bacteriophage inspired growth-decoupled recombinant protein production [18], and by the ability to induce lac derived P_T7_ promoter controlled GOI by addition of different concentrations of l-arabinose, we are able to control expression rate on population-level over a broad range and simultaneously enhance heterologous MP production by reallocation of resources through decoupling growth from RPP. Furthermore, we show for the first time that the effect of all-or-none induction on a population level in P_araBAD_ derived expression systems can be overcome by pulse induction of l-arabinose when used as a combined inducer for P_araBAD_ controlled T7 RNAP and P_T7-lacO_-controlled GOI. By this approach, we can reduce heterogeneity in cellular populations upon the tuning of recombinant protein production to a minimum and thereby allow for significant reduction or even prevention of inclusion body formation.

Our approach allows for the precise transcriptional regulation of protein expression rates on a cellular level. Protein expression can be fine-tuned in a concentration-dependent manner by using a broad range of l-arabinose concentrations without the requirement of additional plasmid or genetic engineering efforts and simultaneously allow for the reallocation of resources due to l-arabinose induced growth decoupling by phage derived inhibitor peptide Gp2 [18]. This benefits expression studies of membrane proteins or other difficult-to-express proteins that have not yet been tested in a growth-decoupled protein production mode.

We have successfully characterized the system under relevant fed-batch like conditions in microscale (800 µL) and generated a data set showing a significant increase in specific yields for 6 different *E. coli* derived MP-GFP fusion proteins. In all cases, the tested BL21-AI<*gp2*>outperformed the parent strain BL21-AI (operated in growth-associated production mode). Specific yields have been improved up to 2.7-fold.

In further studies, we will aim at elucidating the transcriptional response of the described host cell chassis to improve our understanding of the beneficial effects of Gp2 co-expression on recombinant protein and bio-based chemical production. We also expect the improved growth decoupled production strain BL21-AI<*gp2*> to be favorable for the production of metabolites where expression rate control of multiple enzyme pathways is required to allow for maximum possible product fluxes.

## Methods

### Bacterial strains

Experiments in this study were performed with *E. coli* strains BL21(DE3) (F^−^ ompT gal dcm lon hsdSB(rB^−^ mB^−^) λ(DE3 [lacI lacUV5-T7 gene 1 ind1 sam7 nin5]) (Coli Genetic Stock Center #12504) and BL21-AI (F^–^ ompT gal dcm lon hsdS_B_(r_B_^–^m_B_^–^) [malB^+^]_K-12_(λ^S^) araB::T7RNAP-tetA)) (ThermoFisher Scientific) which was used as reference system.

BL21-AI<*gp2*> was generated by linear double-stranded DNA(dsDNA) cartridges, containing the expression unit from pROCOLI(*gp2*) fused to a chloramphenicol acetyltransferase resistance gene (CatR), which were integrated into the bacterial chromo- some at the *att*Tn7 site of *E. coli* BL21-AI according to Stargardt et al. [18]. Since BL21-AI already possess a knockout of the *araB* gene, by that inhibiting metabolization of inducer l-arabinose, no further knockout of the l-arabinose-operon were performed.

### Plasmids

Creation of pET30a(GFPmut3.1)cer is described elsewhere [18]. MP-GFP fusion proteins (YhdY-GFP [70, 71, 75], Psta-GFP [68, 76–78], YliF-GFP [66, 79–82], Ydik-GFP [83], YhhJ-GFP [66, 70, 71] and YfbF-GFP [17, 66, 84]) were established in pET28a vector backbone. MP-GFP fusion proteins have been designed according to Daley et al. and dsDNA was synthesized [66]. Briefly, plasmids contain the MP of interest, followed by a linker sequence encoding a TEV protease site (TCGGTACCTGGATCCGAAAACCTGTACTTCCAGGGTCAATTC), followed by the *gfp* gene (S65T, F64L + Cycle 3mutant) and an 8xHis-tag at the 3′ end. All genes are preceded by the same ribosome binding site (AGGAGA) and the start codon is always ATG. All constructs were confirmed by Sanger sequencing.

### Medium, cultivation conditions and sampling

The strains were cultivated in duplicates in the BioLector micro-fermentation system, in 48-well Flowerplates® (m2p-labs) as described by Toeroek et al. [85]. All cultivations were carried out in enzymatic (1% (v/v) Enzymix, m2p-labs GmbH, Baesweiler, Germany) glucose release media (65% (v/v) FIT fed-batch medium, m2p-labs GmbH, Baesweiler, Germany), containing D-glucose (1% (v/v)) as carbon source for the batch-phase and dextran, which is enzymatically converted to D-glucose, for the subsequent feeding-phase*.* CDM concentration was calculated from scattered light signal by linear regression analysis where scattered light signal of different diluted CDM concentrations was correlated to gravimetrically determined CDM samples from strain BL21(DE3), BL21-AI and BL21-AI<*gp2*> . Expression level of green fluorescence protein was monitored by an excitation wavelength of 488 nm and emission wavelength of 520 nm. All experiments were carried out at 30 °C and lasted 24 h. The cycle time for all online measurements was 15 min. The shaking frequency was set to 1400 rpm and humidity level in the cultivation chamber was controlled at a level above 80%. The working volume in each well was 800 µL. Inoculation was performed with a density which was equivalent to an optical density of OD_600_ = 0.2. Inoculation process is described elsewhere [85]. One well with medium was used for sterility control. Recombinant gene expression in *E. coli* strains BL21(DE3), BL21-AI and BL21-AI<*gp2*> harboring the different plasmids were induced with different concentrations of IPTG (GERBU Biotechnik, Germany), l-arabinose (Merck KGaA, Germany) or combined induction of both inducers. Induction started approximately 13 h after inoculation of the process.

### Offline analysis

#### Flow cytometry analysis

Gallios flow cytometer (Beckman Coulter, USA) was used to quantify populations of GFPmut3.1 or MP-GFP fusion proteins—producing cells. Sampling took place 8 h after induction and measurement was performed according to Schuller et al. [55]. MFI was calculated by geometric mean of FL1-A signal of induced cells. Cell size was estimated by geometric mean calculation of FSC-A signal of whole cell population. Experiments were performed in duplicate.

#### SDS-Page analysis

Cell disintegration and protein extraction was performed according to Fink et al. [86]. For extraction NuPAGE® Sample Reducing Agent (10X) (Novex) was additionally added to a concentration of 4 mM to the lysis buffer, respectively. Proteins of interest were detected on SDS–polyacrylamide electrophoresis (PAGE) gel (Invitrogen NuPAGE® 4–12% Bis–Tris) according to Stargardt et al. [18].

#### Western Blot analysis

Soluble MP-GFP expression, IB formation, and basal expression levels were analyzed with WBs according to Fink et al. [86]. For extraction NuPAGE® Sample Reducing Agent (10X) (Novex) was additionally added to a concentration of 4 mM to the lysis buffer, respectively. MP-GFP proteins was captured with Anti-GFP monoclonal antibody (Sigma-Aldrich, G6539) and detected with alkaline phosphatase‐labeled anti‐mouse IgG (whole molecule) (A5153; Sigma‐Aldrich).

## Supplementary Information

**Additional file 1: Figure S1.** Protein sequence of T7 RNA-polymerase found in strain BL21(DE3) compared to sequence found in strain BL21-AI. **Figure S2.** Flow cytometry analysis of single‑cell expression of GFPmut3.1 by strain BL21-AI<*gp2*> during fed-batch like cultivation. **Figure S3.** Flow cytometry analysis of single‑cell expression of GFPmut3.1 by strain BL21-AI<*gp2*> during fed-batch like cultivation. **Figure S4.** Flow cytometry analysis of single‑cell expression of GFPmut3.1 by strain BL21-AI during fed-batch like cultivation. **Figure S5.** Flow cytometry analysis of single‑cell expression of GFPmut3.1 by strain BL21-AI during fed-batch like cultivation. **Figure S6.** Flow cytometry analysis of single‑cell expression of GFPmut3.1 by strain BL21-AI<*gp2*> during fed-batch like cultivation. **Figure S7.** Flow cytometry analysis of single‑cell expression of GFPmut3.1 by strain BL21-AI<*gp2*> during fed-batch like cultivation. **Figure S8.** Flow cytometry analysis of single‑cell expression of GFPmut3.1 by strain BL21-AI during fed-batch like cultivation. **Figure S9.** Flow cytometry analysis of single‑cell expression of GFPmut3.1 by strain BL21-AI during fed-batch like cultivation. **Figure S10.** Flow cytometry analysis of strain BL21-AI, harboring no reporting plasmid, during fed-batch like cultivation. **Figure S11.** Product formation kinetics and flow cytometry analysis of single‑cell expression of *E. coli* strains BL21-AI (**a**, **b**, **c**, **g**, **h**, **i**) and BL21-AI<*gp2*> (**d**, **e**, **f**, **j**, **k**, **l**) expressing Yhdy-GFP fusion protein during fed-batch like cultivation. **Figure S12.** Process characteristic showing product formation kinetics and flow cytometry analysis of *E. coli* strains BL21-AI (**a**, **b**, **c**) and BL21-AI<*gp2*> (**d**, **e**, **f**) expressing Yhdy-GFP fusion protein during fed-batch like cultivation. Induction was performed with 100 mM l-arabinose and 100 mM l-arabinose + 1 mM IPTG. The mean CDM [mg] and mean specific GFP yield [rfu/mg] represents duplicate samples. **Figure S13.** Product formation kinetics and flow cytometry analysis of single‑cell expression of *E. coli* strains BL21-AI (**a**, **b**, **c**, **g**, **h**, **i**) and BL21-AI<*gp2*> (**d**, **e**, **f**, **j**, **k**, **l**) expressing PstA-GFP fusion protein during fed-batch like cultivation. **Figure S14.** Process characteristic showing product formation kinetics and flow cytometry analysis of *E. coli* strains BL21-AI (**a**, **b**, **c**) and BL21-AI<*gp2*> (**d**, **e**, **f**) expressing PstA-GFP fusion protein during fed-batch like cultivation. **Figure S15.** Product formation kinetics and flow cytometry analysis of single‑cell expression of E. coli strains BL21-AI (**a**, **b**, **c**, **g**, **h**, **i**) and BL21-AI<*gp2*>  (**d**, **e**, **f**, **j**, **k**, **l**) expressing YliF-GFP fusion protein during fed-batch like cultivation. **Figure S16.** Process characteristic showing product formation kinetics and flow cytometry analysis of *E. coli* strains BL21-AI (**a**, **b**, **c**) and BL21-AI<*gp2*> (** d**, **e**, **f**) expressing YliF-GFP fusion protein during fed-batch like cultivation. **Figure S17.** Product formation kinetics and flow cytometry analysis of single‑cell expression of *E. coli* strains BL21-AI (**a**, **b**, **c**, **g**, **h**, **i**) and BL21-AI<*gp2*> (**d**, **e**, **f**, **j**, **k**, **l**) expressing YdiK-GFP fusion protein during fed-batch like cultivation. **Figure S18.** Process characteristic showing product formation kinetics and flow cytometry analysis of *E. coli* strains BL21-AI (**a**, **b**, **c**) and BL21-AI<*gp2*> (**d**, **e**, **f**) expressing YdiK-GFP fusion protein during fed-batch like cultivation. **Figure S19.** Product formation kinetics and flow cytometry analysis of single‑cell expression of *E. coli* strains BL21-AI (**a**, **b**, **c**, **g**, **h**, **i**) and BL21-AI<*gp2*> (** d**, **e**, **f**, **j**, **k**, **l**) expressing YhhJ-GFP fusion protein during fed-batch like cultivation. **Figure S20.** Process characteristic showing product formation kinetics and flow cytometry analysis of *E. coli* strains BL21-AI (**a**, **b**, **c**) and BL21-AI<*gp2*> (**d**, **e**, **f**) expressing YhhJ-GFP fusion protein during fed-batch like cultivation. **Figure S21.** Product formation kinetics and flow cytometry analysis of single‑cell expression of *E. coli* strains BL21-AI (**a**, **b**, **c**, **g**, **h**, **i**) and BL21-AI<*gp2*> (**d**, **e**, **f**, **j**, **k**, **l**) expressing YfbF-GFP fusion protein during fed-batch like cultivation. **Figure S22.** Process characteristic showing product formation kinetics and flow cytometry analysis of *E. coli* strains BL21-AI (**a**, **b**, **c**) and BL21-AI<*gp2*> (**d**, **e**, **f**) expressing YfbF-GFP fusion protein during fed-batch like cultivation. **Figure S23.** Western Blot showing overexpression of YhdY-GFP during fed-batch cultivations of *E. coli* strain BL21-AIand BL21-AI<*gp2*> . **Figure S24.** Western Blot showing overexpression of PstA-GFP during fed-batch cultivations of *E. coli* strain BL21-AI and BL21-AI<*gp2*> . **Figure S25.** Western Blot showing overexpression of YliF-GFP during fed-batch cultivations of *E. coli* strain BL21-AI and BL21-AI<*gp2*> . **Figure S26.** Western Blot showing overexpression of YdiK-GFP during fed-batch cultivations of *E. coli* strain BL21-AI and BL21-AI<*gp2*> . **Figure S27.** Western Blot showing overexpression of YfbF-GFP during fed-batch cultivations of *E. coli* strain BL21-AI and BL21-AI<*gp2*> . **Figure S28.** Western Blot showing overexpression of YhhJ-GFP during fed-batch cultivations of *E. coli* strain BL21-AI and BL21-AI<*gp2*>.

## Abbreviations

CatR: Chloramphenicol acetyltransferase resistance gene
CDM: Cell dry mass
*E. coli*: *Escherichia coli*
GFP: Green fluorescent protein
GOI: Gene of interest
Gp2: Gene product 2
IPTG: Isopropyl β-d-1-thiogalactopyranoside
KanR: Kanamycin resistance gene
MFI: Mean fluorescence intensity
MP: Membrane protein
PAC: Penicillin G acylase
POI: Protein of interest
RNAP: RNA polymerase
RPP: Recombinant protein production
