# Supplementary material for: Tunable expression rate control of a growth-decoupled T7 expression system by l-arabinose only
Source: Microb Cell Fact. 2021 Feb 1;20:27. doi: 10.1186/s12934-021-01512-7 (PMC7852362; doi:10.1186/s12934-021-01512-7)
Supplement: Supplementary file 1 — Additional file 1: Figure S1. Protein sequence of T7 RNA-polymerase found in strain BL21(DE3) compared to sequence found in strain BL21-AI. Figure S2. Flow cytometry analysis of single‑cell expression of GFPmut3.1 by strain BL21-AI during fed-batch like cultivation. Figure S3. Flow cytometry analysis of single‑cell expression of GFPmut3.1 by strain BL21-AI during fed-batch like cultivation. Figure S4. Flow cytometry analysis of single‑cell expression of GFPmut3.1 by strain BL21-AI during fed-batch like cultivation. Figure S5. Flow cytometry analysis of single‑cell expression of GFPmut3.1 by strain BL21-AI during fed-batch like cultivation. Figure S6. Flow cytometry analysis of single‑cell expression of GFPmut3.1 by strain BL21-AI during fed-batch like cultivation. Figure S7. Flow cytometry analysis of single‑cell expression of GFPmut3.1 by strain BL21-AI during fed-batch like cultivation. Figure S8. Flow cytometry analysis of single‑cell expression of GFPmut3.1 by strain BL21-AI during fed-batch like cultivation. Figure S9. Flow cytometry analysis of single‑cell expression of GFPmut3.1 by strain BL21-AI during fed-batch like cultivation. Figure S10. Flow cytometry analysis of strain BL21-AI, harboring no reporting plasmid, during fed-batch like cultivation. Figure S11. Product formation kinetics and flow cytometry analysis of single‑cell expression of E. coli strains BL21-AI (a, b, c, g, h, i) and BL21-AI (d, e, f, j, k, l) expressing Yhdy-GFP fusion protein during fed-batch like cultivation. Figure S12. Process characteristic showing product formation kinetics and flow cytometry analysis of E. coli strains BL21-AI (a, b, c) and BL21-AI (d, e, f) expressing Yhdy-GFP fusion protein during fed-batch like cultivation. Induction was performed with 100 mM l-arabinose and 100 mM l-arabinose + 1 mM IPTG. The mean CDM [mg] and mean specific GFP yield [rfu/mg] represents duplicate samples. Figure S13. Product formation kinetics and flow cytometr [file 12934_2021_1512_MOESM1_ESM.docx]

# Supplementary


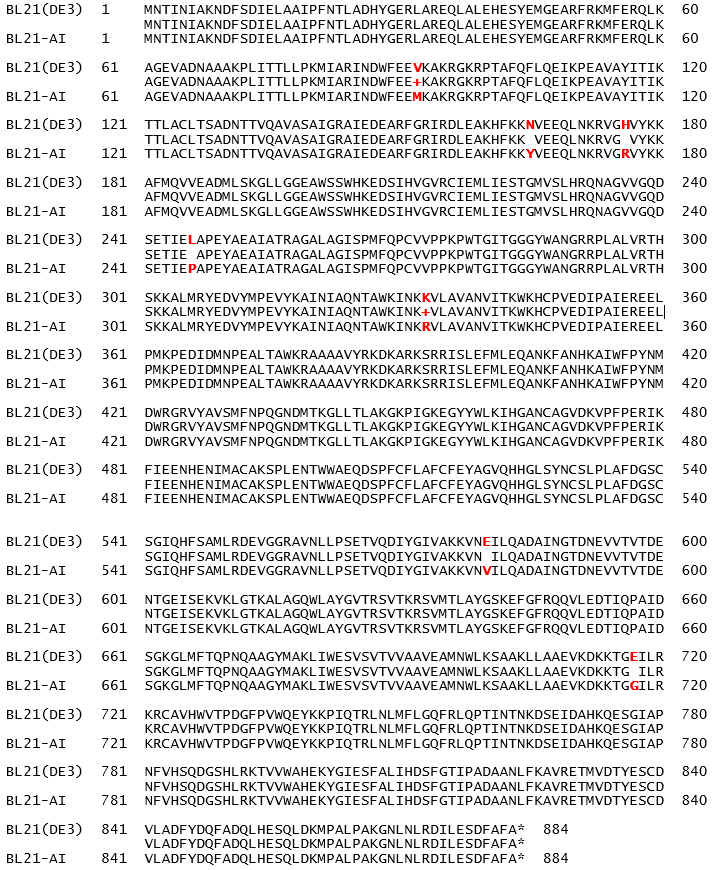


Supplementary Figure 1 Protein sequence of T7 RNA-polymerase found in strain BL21(DE3) compared to sequence found in strain BL21-AI. Mutations in the T7 RNA-polymerase sequence are shown in red letters.


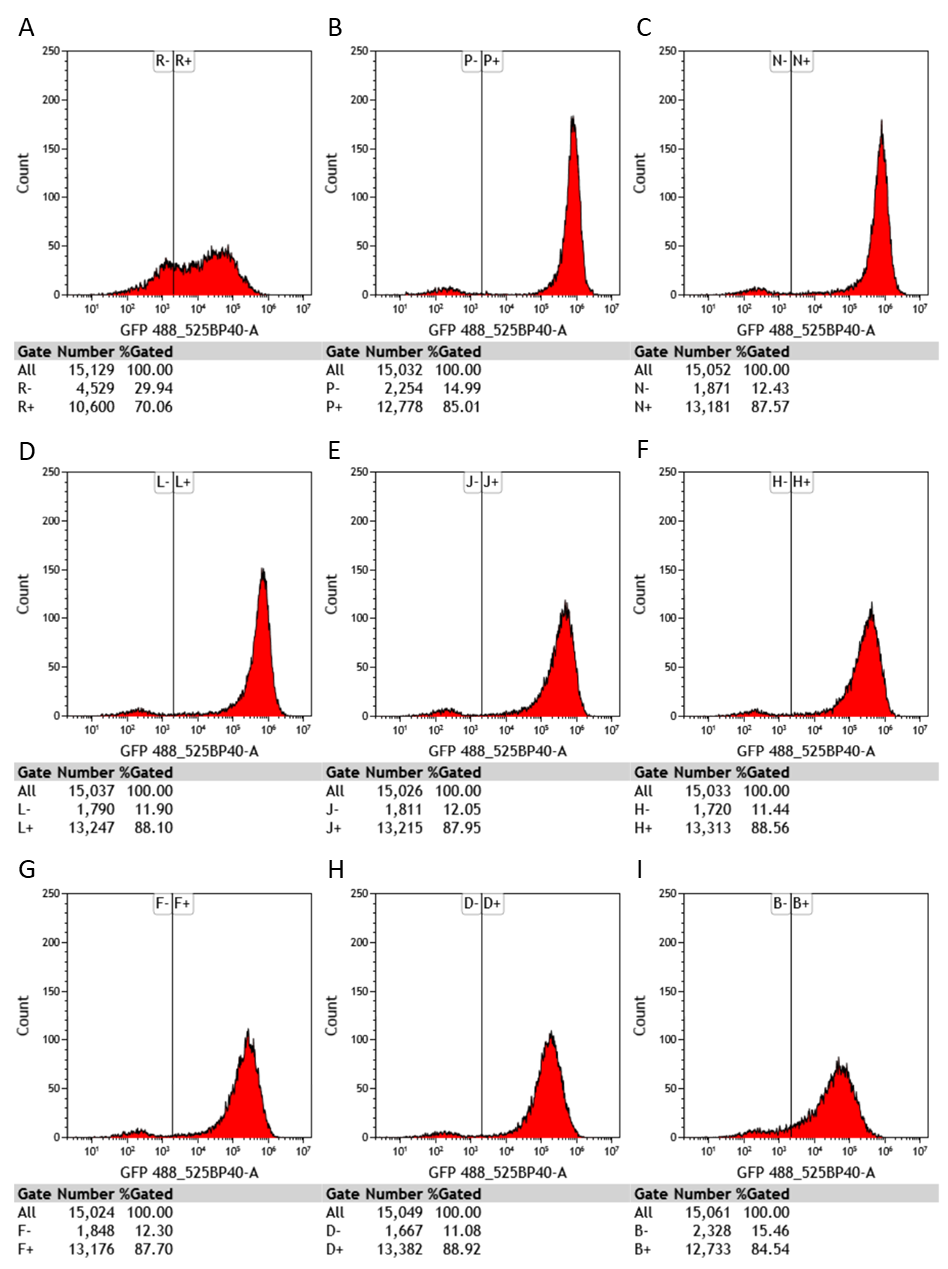


Supplementary Figure 2 Flow cytometry analysis of single‑cell expression of GFPmut3.1 by strain BL21-AI<gp2> during fed-batch like cultivation. Non-induced cultivation is shown in (A). Induction was performed with 100 mM L-arabinose + 1 mM IPTG (B), 100 mM (C), 25 mM (D), 5 mM (E), 2.5 mM (F), 1 mM (G), 0.25 mM (H) or 0.025 mM (I) L-arabinose. Cultivations were performed with 50 µg/mL Kanamycin. Experiments were performed in duplicate. Results from a single experiment are presented.


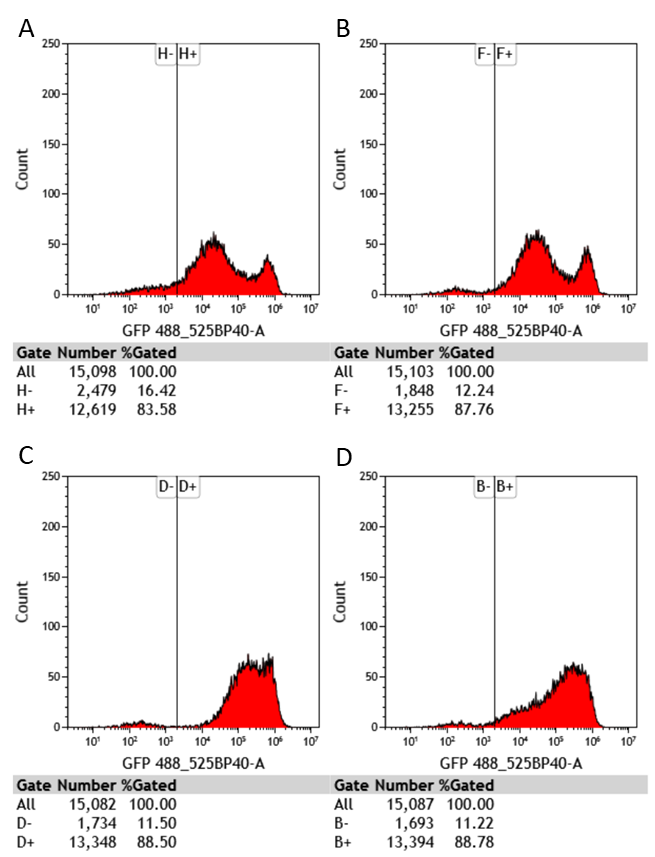


Supplementary Figure 3 Flow cytometry analysis of single‑cell expression of GFPmut3.1 by strain BL21-AI<gp2> during fed-batch like cultivation. Induction was performed with 10 mM (A), 1 mM (B), 0.1 mM (C) or 0.01 mM (D) IPTG. Cultivations were performed with 50 µg/mL Kanamycin. Experiments were performed in duplicate. Results from a single experiment are presented.


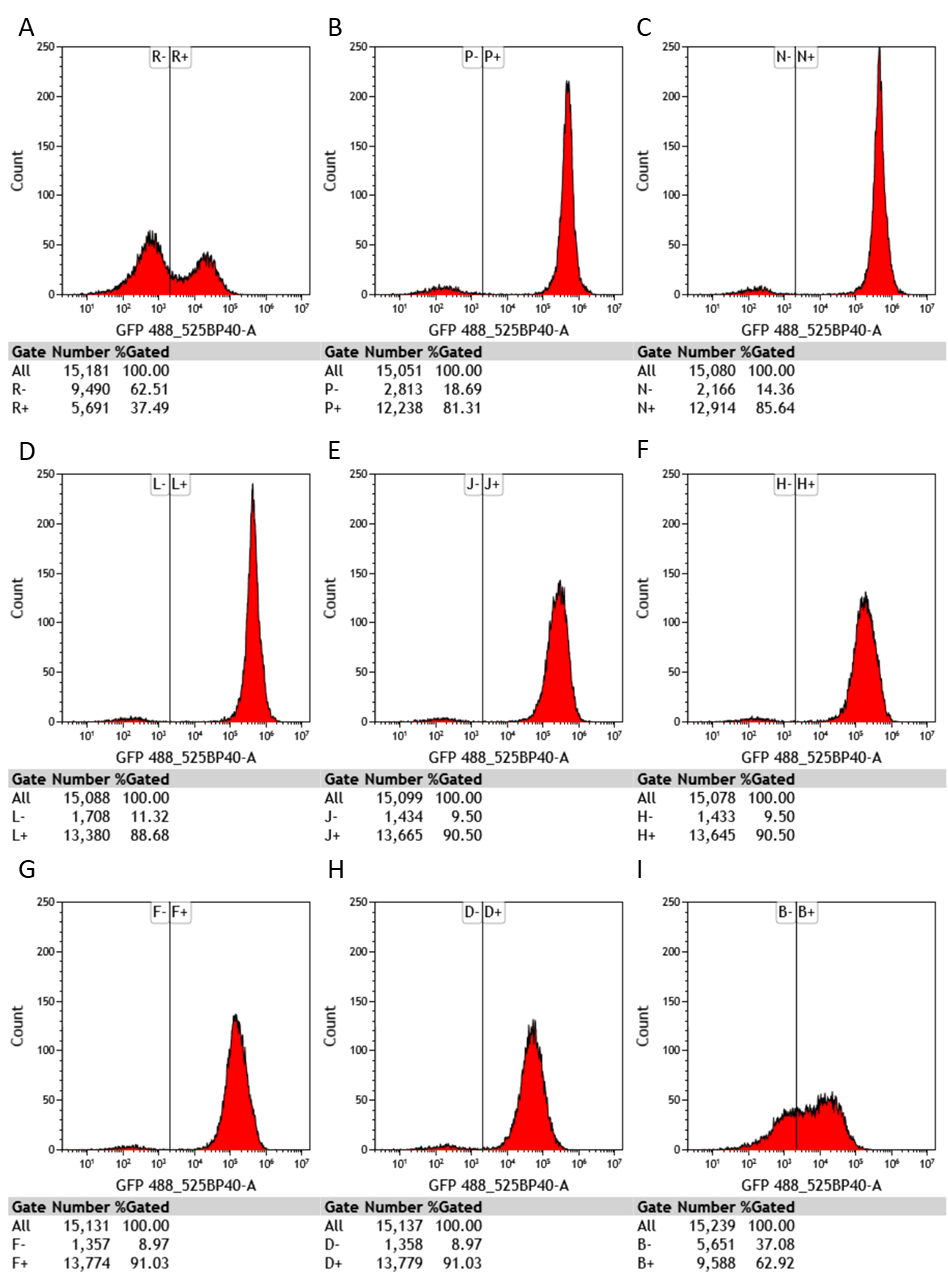


Supplementary Figure 4 Flow cytometry analysis of single‑cell expression of GFPmut3.1 by strain BL21-AI during fed-batch like cultivation. Non-induced cultivation is shown in (A). Induction was performed with 100 mM L-arabinose + 1 mM IPTG (B), 100 mM (C), 25 mM (D), 5 mM (E), 2.5 mM (F), 1 mM (G), 0.25 mM (H) or 0.025 mM (I) L-arabinose. Cultivations were performed with 50 µg/mL Kanamycin. Experiments were performed in duplicate. Results from a single experiment are presented.


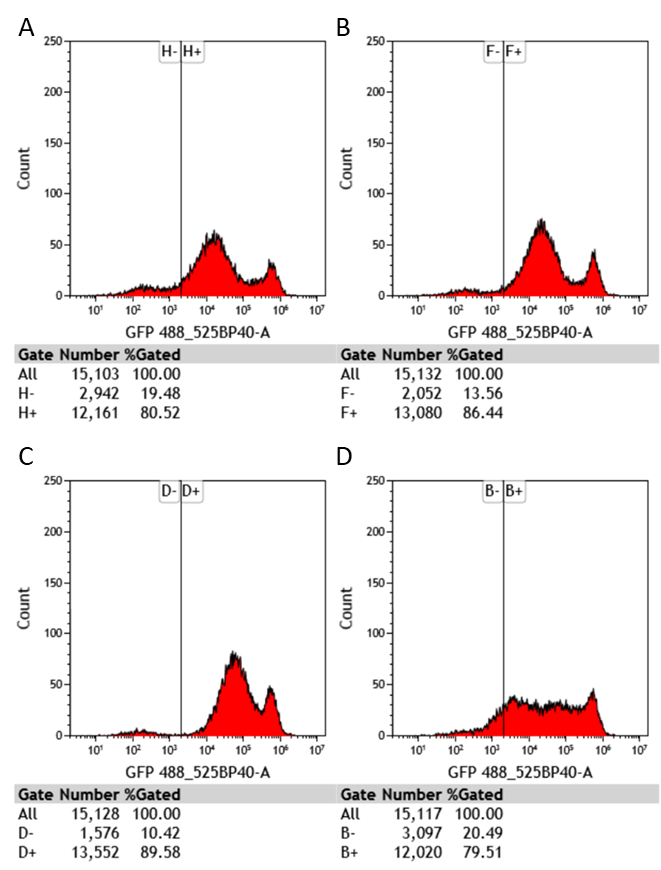


Supplementary Figure 5 Flow cytometry analysis of single‑cell expression of GFPmut3.1 by strain BL21-AI during fed-batch like cultivation. Induction was performed with 10 mM (A), 1 mM (B), 0.1 mM (C) or 0.01 mM (D) IPTG. Cultivations were performed with 50 µg/mL Kanamycin. Experiments were performed in duplicate. Results from a single experiment are presented.


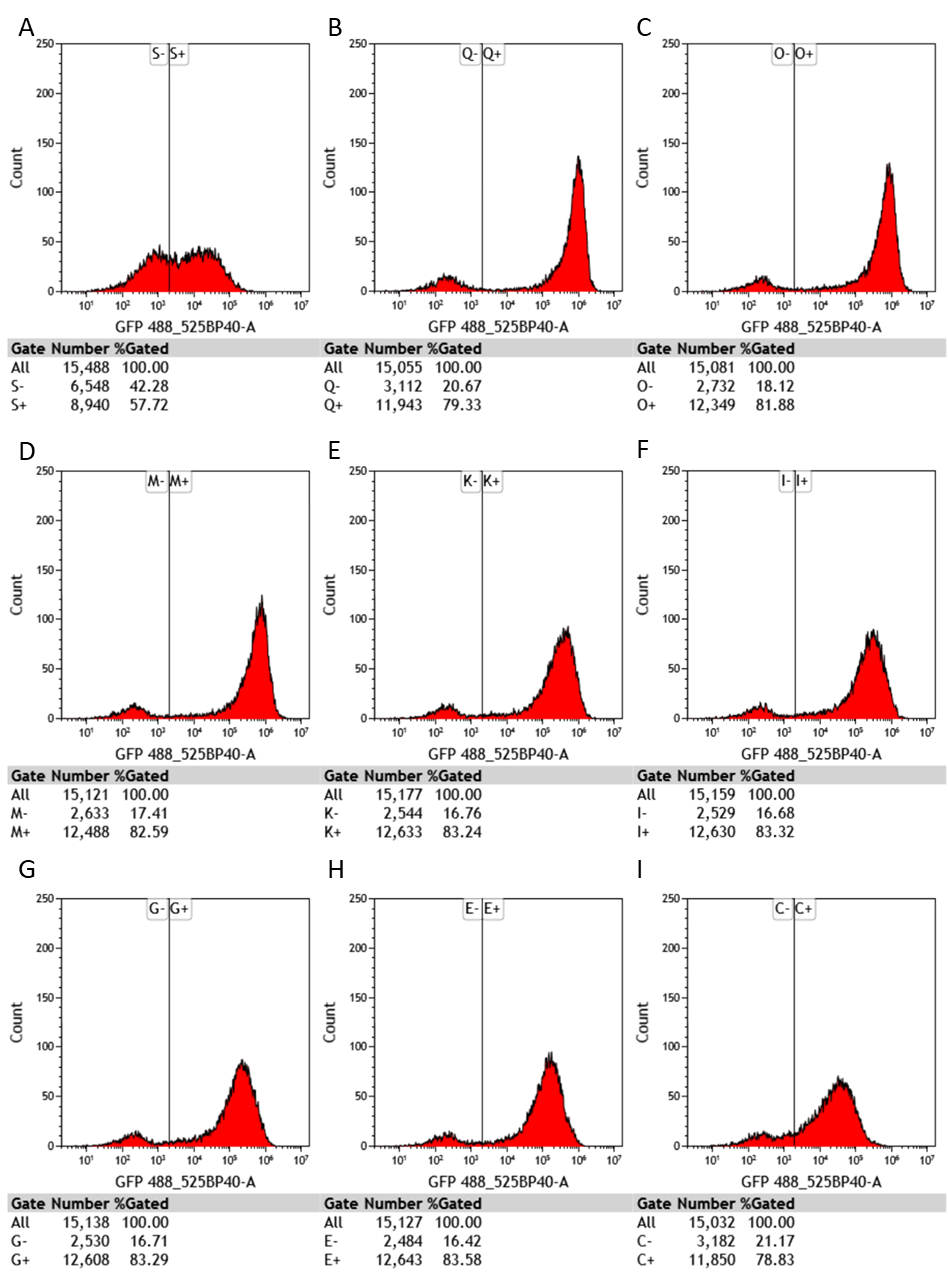


Supplementary Figure 6 Flow cytometry analysis of single‑cell expression of GFPmut3.1 by strain BL21-AI<gp2> during fed-batch like cultivation. Non-induced cultivation is shown in (A). Induction was performed with 100 mM L-arabinose + 1 mM IPTG (B), 100 mM (C), 25 mM (D), 5 mM (E), 2.5 mM (F), 1 mM (G), 0.25 mM (H) or 0.025 mM (I) L-arabinose. Cultivations were performed without antibiotics. Experiments were performed in duplicate. Results from a single experiment are presented.


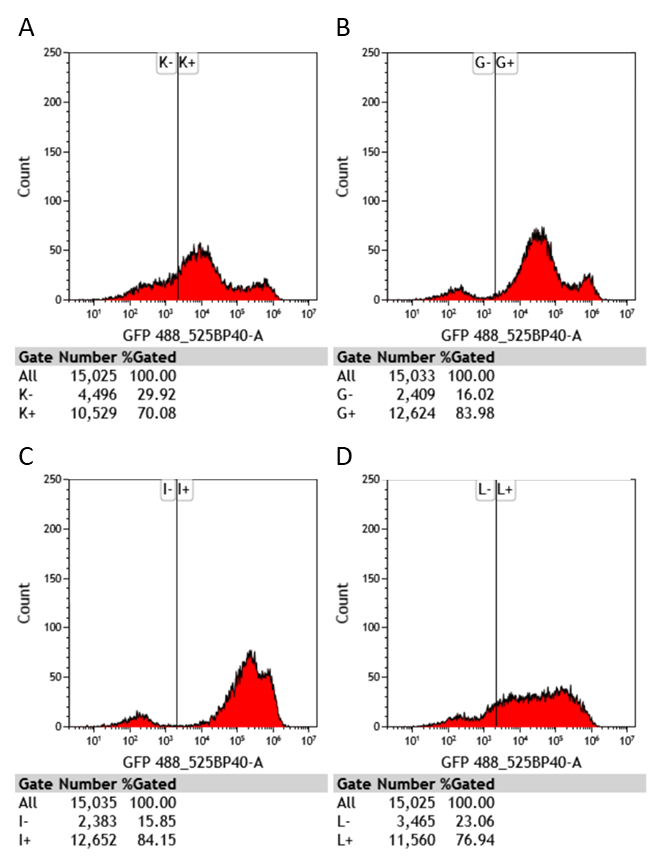


Supplementary Figure 7 Flow cytometry analysis of single‑cell expression of GFPmut3.1 by strain BL21-AI<gp2> during fed-batch like cultivation. Induction was performed with 10 mM (A), 1 mM (B), 0.1 mM (C) or 0.01 mM (D) IPTG. Cultivations were performed without antibiotics. Experiments were performed in duplicate. Results from a single experiment are presented.


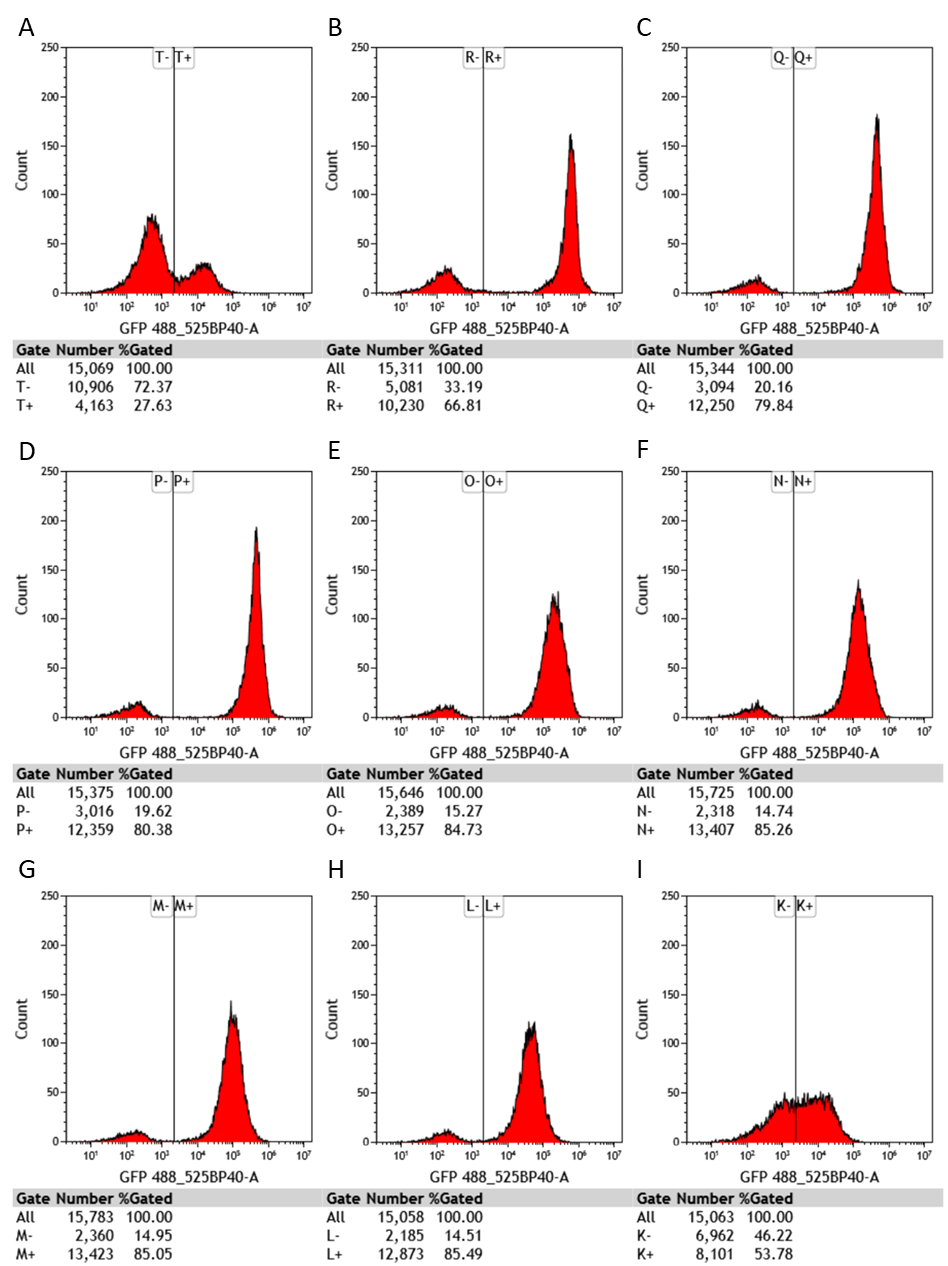


Supplementary Figure 8 Flow cytometry analysis of single‑cell expression of GFPmut3.1 by strain BL21-AI during fed-batch like cultivation. Non-induced cultivation is shown in (A). Induction was performed with 100 mM L-arabinose + 1 mM IPTG (B), 100 mM (C), 25 mM (D), 5 mM (E), 2.5 mM (F), 1 mM (G), 0.25 mM (H) or 0.025 mM (I) L-arabinose. Cultivations were performed without antibiotics. Experiments were performed in duplicate. Results from a single experiment are presented.


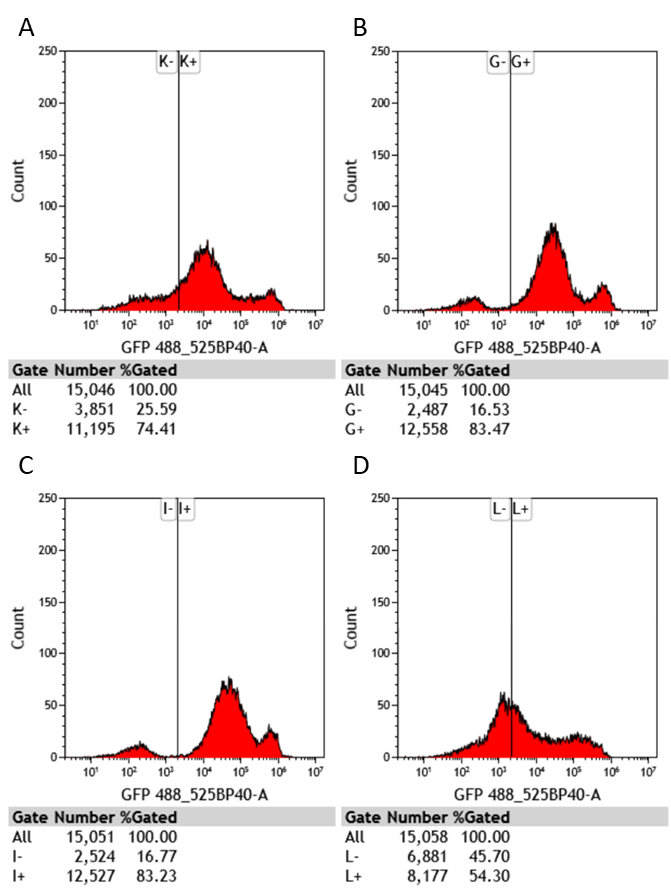


Supplementary Figure 9 Flow cytometry analysis of single‑cell expression of GFPmut3.1 by strain BL21-AI during fed-batch like cultivation. Induction was performed with 10 mM (A), 1 mM (B), 0.1 mM (C) or 0.01 mM (D) IPTG. Cultivations were performed without antibiotics. Experiments were performed in duplicate. Results from a single experiment are presented.


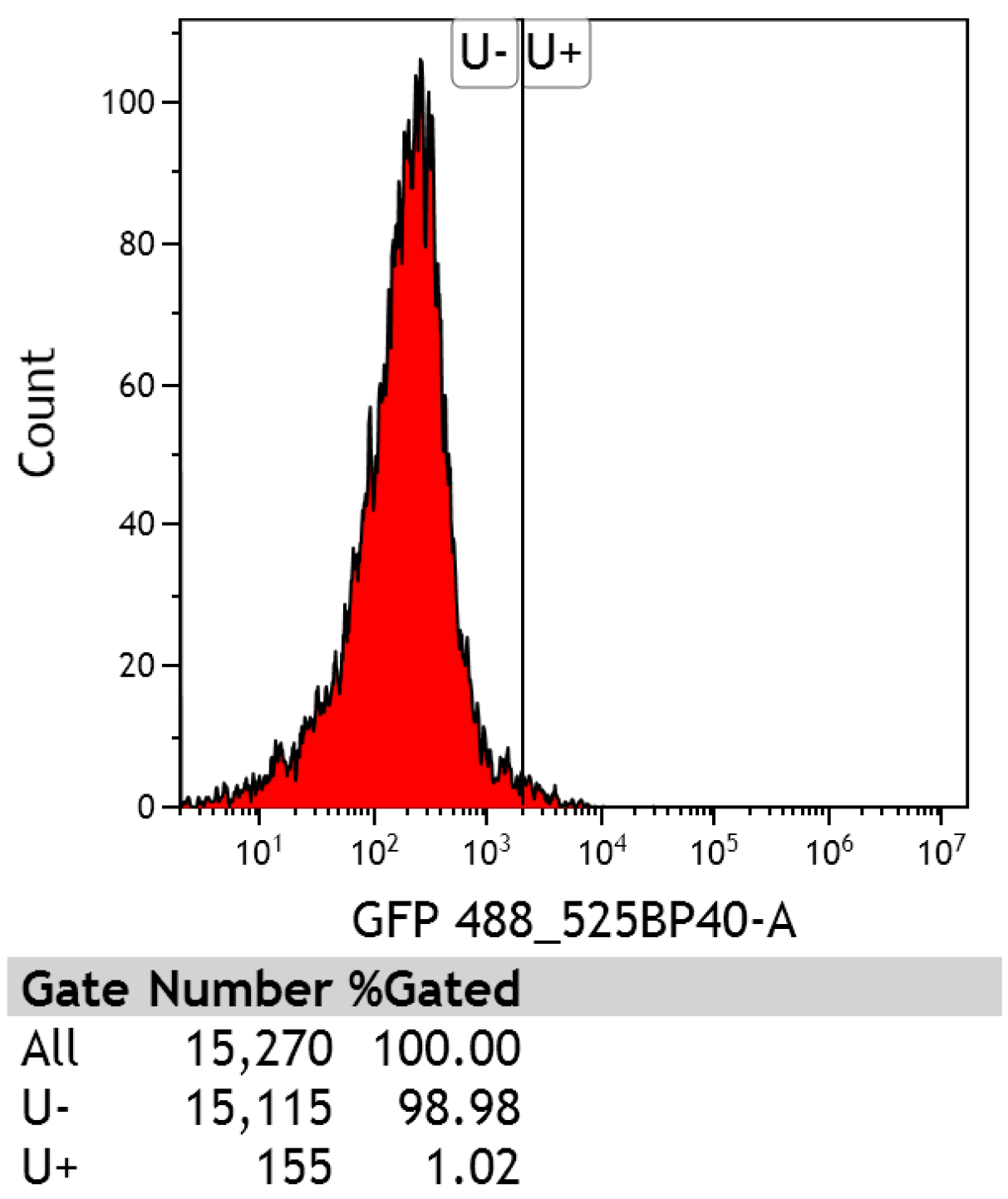


Supplementary Figure 10 Flow cytometry analysis of strain BL21-AI, harboring no reporting plasmid, during fed-batch like cultivation. Cultivations were performed without antibiotics. Experiments were performed in duplicate. Results from a single experiment are presented.


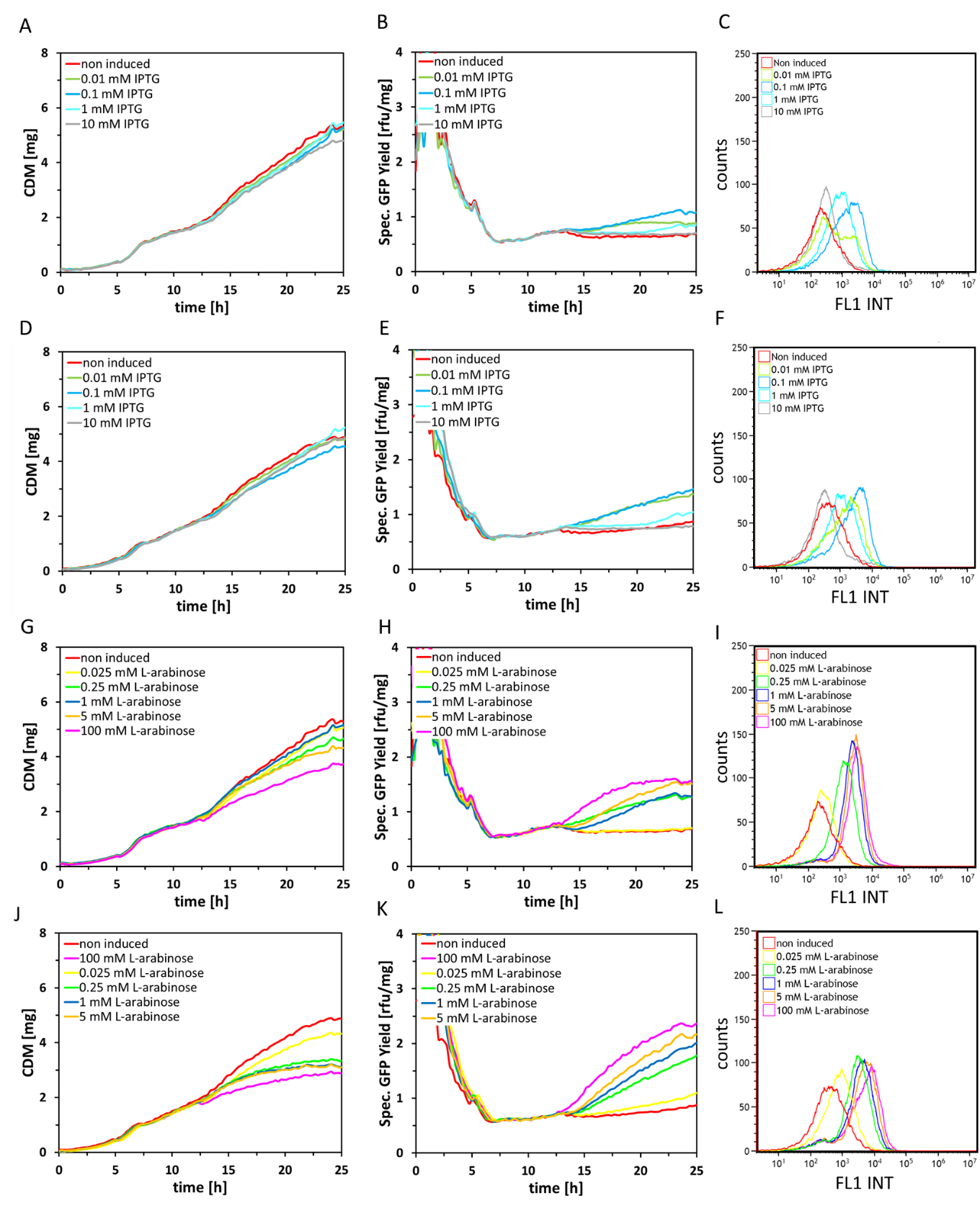


Supplementary Figure 11 Product formation kinetics and flow cytometry analysis of single‑cell expression of E. coli strains BL21-AI (**A, B, C, G, H, I**) and BL21-AI<gp2> (**D, E, F, J, K, L**) expressing Yhdy-GFP fusion protein during fed-batch like cultivation. Induction was performed either with L-arabinose (0.025, 0.25, 1, 5, 100 mM) only (**G, H, I, J, K, L**) or with IPTG (0.01, 0.1, 1, 10 mM) only (**A, B, C, D, E, F**). The mean CDM [mg] and mean specific GFP yield [rfu/mg] represents duplicate samples. Flow cytometry results from a single experiment are presented.


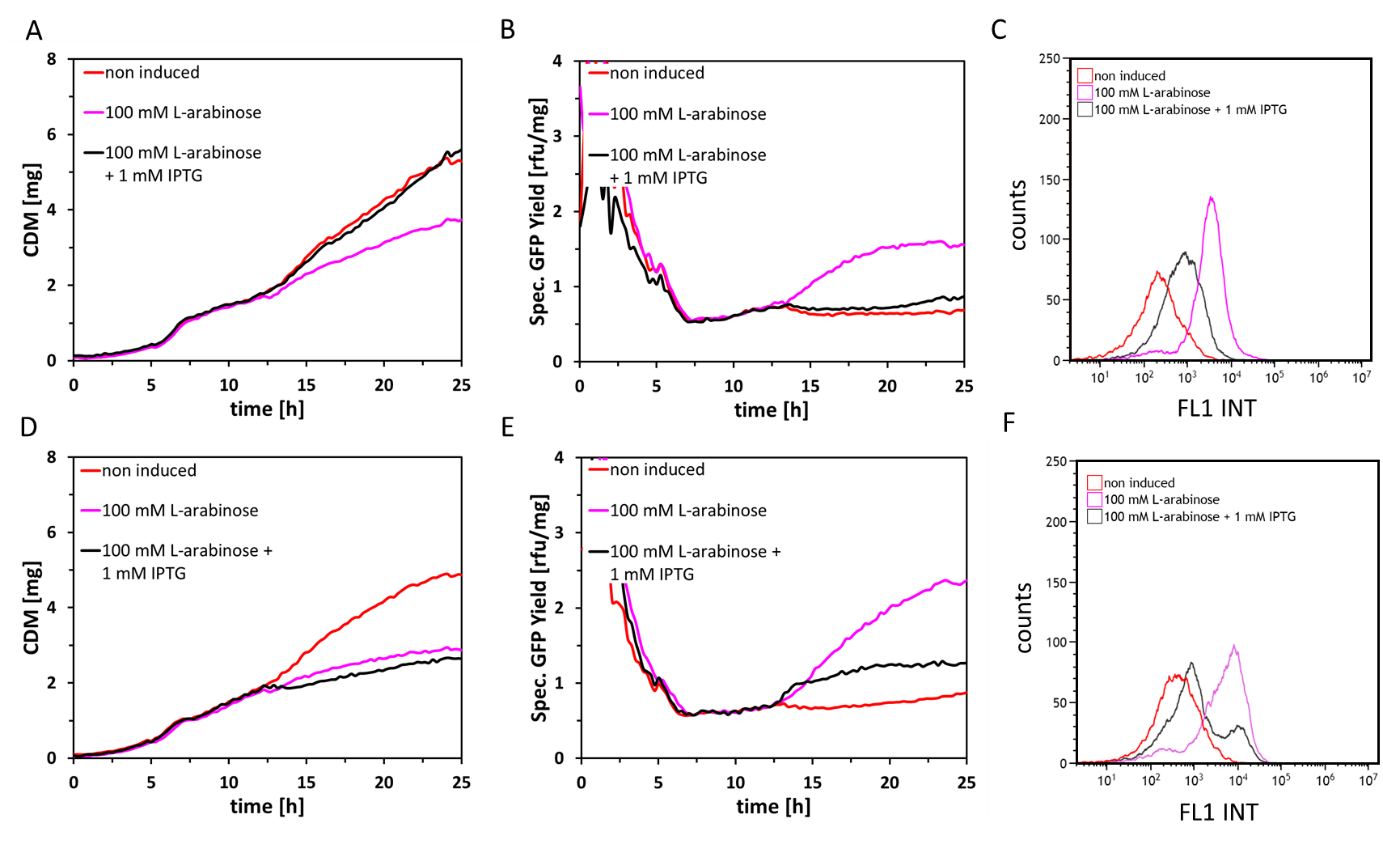


Supplementary Figure 12 Process characteristic showing product formation kinetics and flow cytometry analysis of E. coli strains BL21-AI (**A, B, C**) and BL21-AI<gp2> (**D, E, F**) expressing Yhdy-GFP fusion protein during fed-batch like cultivation. Induction was performed with 100 mM L-arabinose and 100 mM L-arabinose + 1 mM IPTG. The mean CDM [mg] and mean specific GFP yield [rfu/mg] represents duplicate samples. Flow cytometry results from a single experiment are presented.


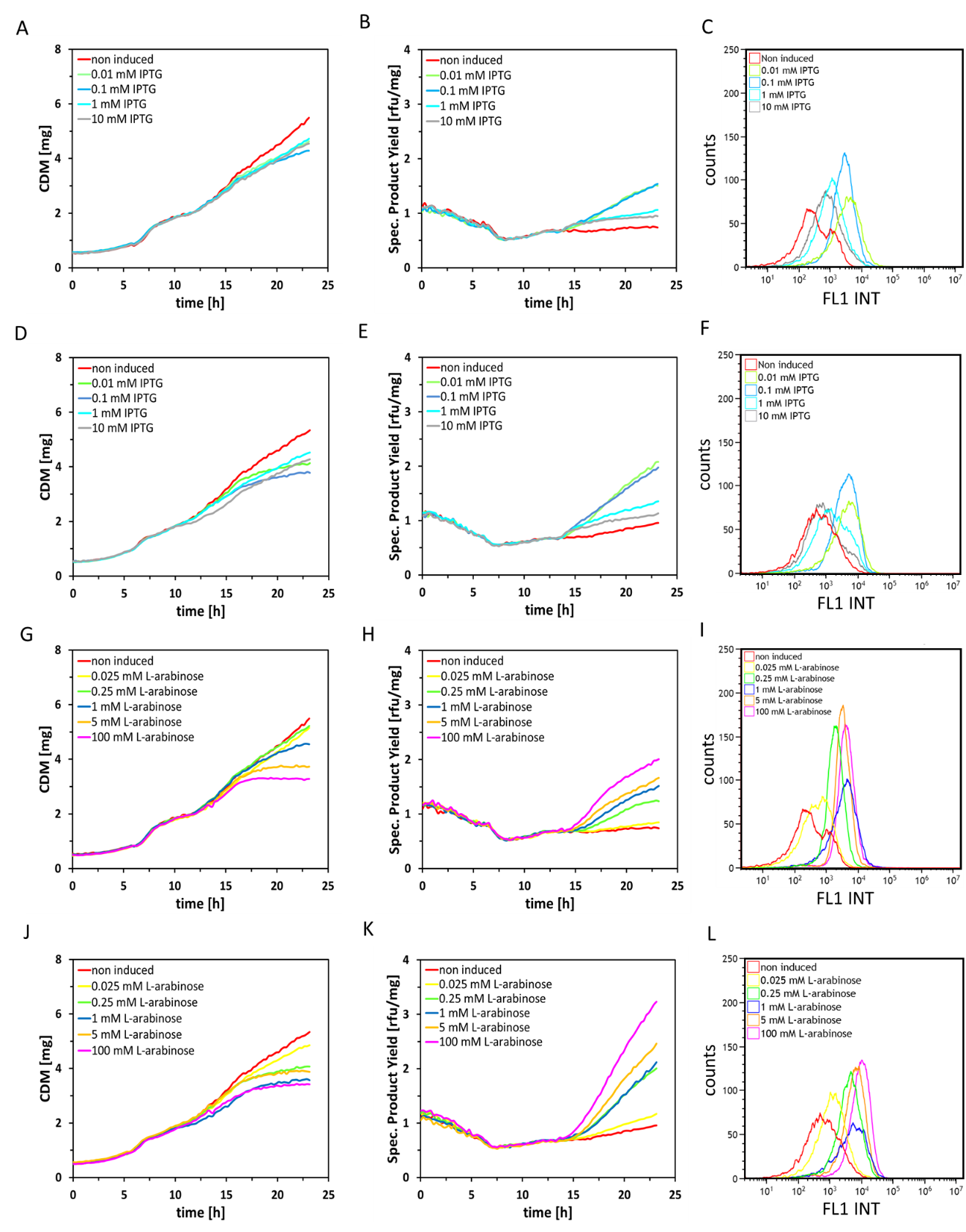


Supplementary Figure 13 Product formation kinetics and flow cytometry analysis of single‑cell expression of E. coli strains BL21-AI (**A, B, C, G, H, I**) and BL21-AI<gp2> (**D, E, F, J, K, L**) expressing PstA-GFP fusion protein during fed-batch like cultivation. Induction was performed either with L-arabinose (0.025, 0.25, 1, 5, 100 mM) only (**G, H, I, J, K, L**) or with IPTG (0.01, 0.1, 1, 10 mM) only (**A, B, C, D, E, F**). The mean CDM [mg] and mean specific GFP yield [rfu/mg] represents duplicate samples. Flow cytometry results from a single experiment are presented.


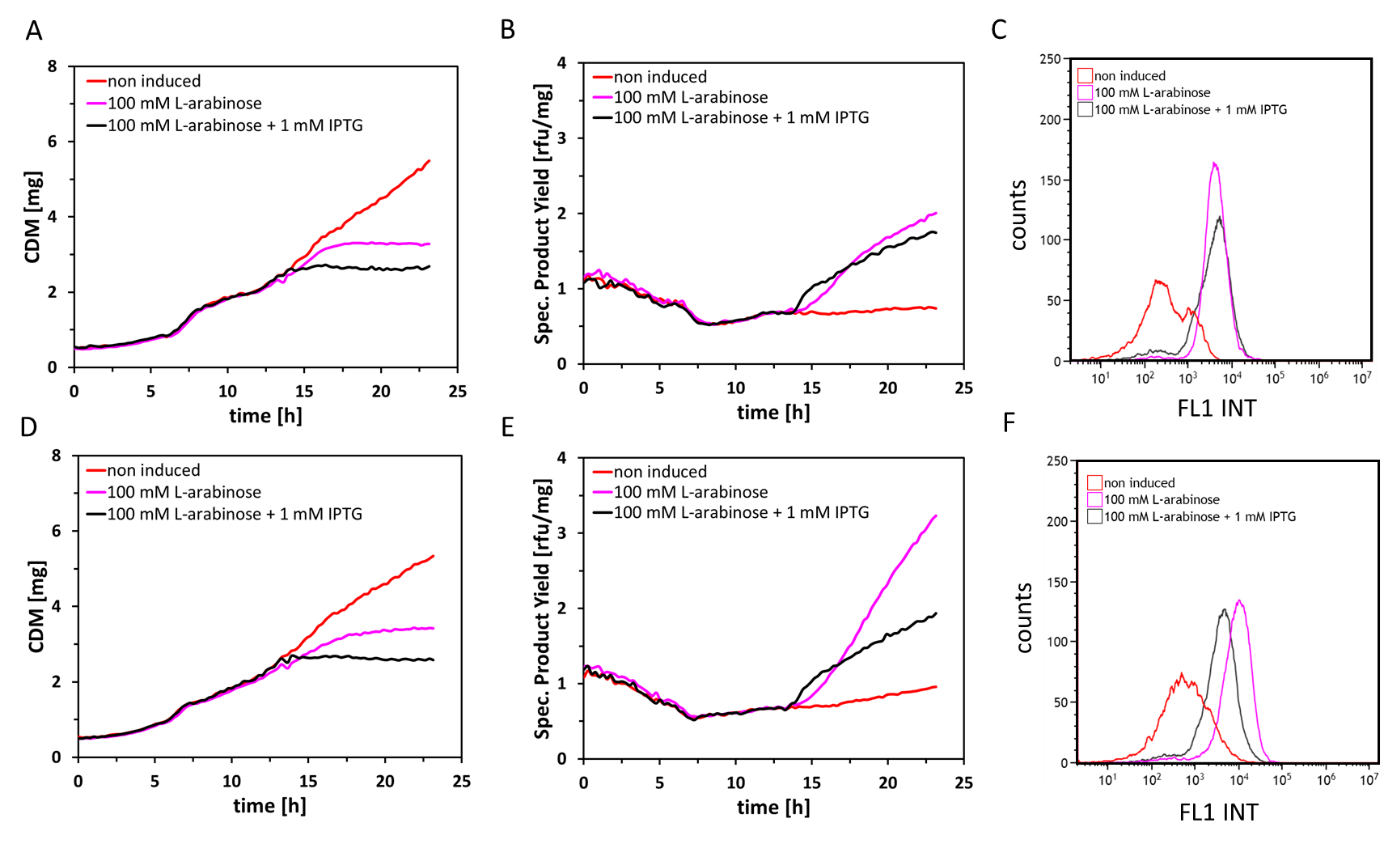


Supplementary Figure 14 Process characteristic showing product formation kinetics and flow cytometry analysis of E. coli strains BL21-AI (**A, B, C**) and BL21-AI<gp2> (**D, E, F**) expressing PstA-GFP fusion protein during fed-batch like cultivation. Induction was performed with 100 mM L-arabinose and 100 mM L-arabinose + 1 mM IPTG. The mean CDM [mg] and mean specific GFP yield [rfu/mg] represents duplicate samples. Flow cytometry results from a single experiment are presented.


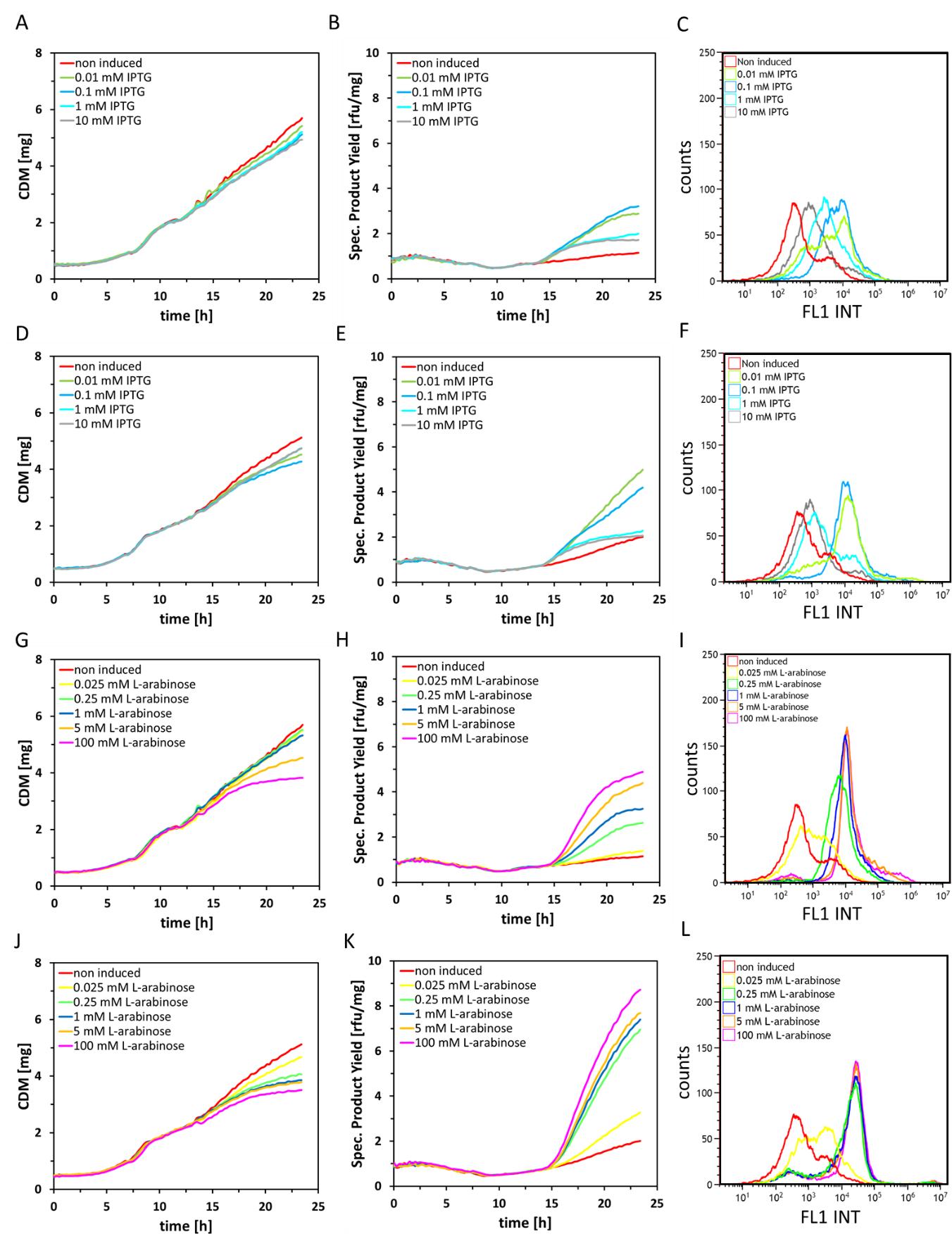


Supplementary Figure 15 Product formation kinetics and flow cytometry analysis of single‑cell expression of E. coli strains BL21-AI (**A, B, C, G, H, I**) and BL21-AI<gp2> (**D, E, F, J, K, L**) expressing YliF-GFP fusion protein during fed-batch like cultivation. Induction was performed either with L-arabinose (0.025, 0.25, 1, 5, 100 mM) only (**G, H, I, J, K, L**) or with IPTG (0.01, 0.1, 1, 10 mM) only (**A, B, C, D, E, F**). The mean CDM [mg] and mean specific GFP yield [rfu/mg] represents duplicate samples. Flow cytometry results from a single experiment are presented.


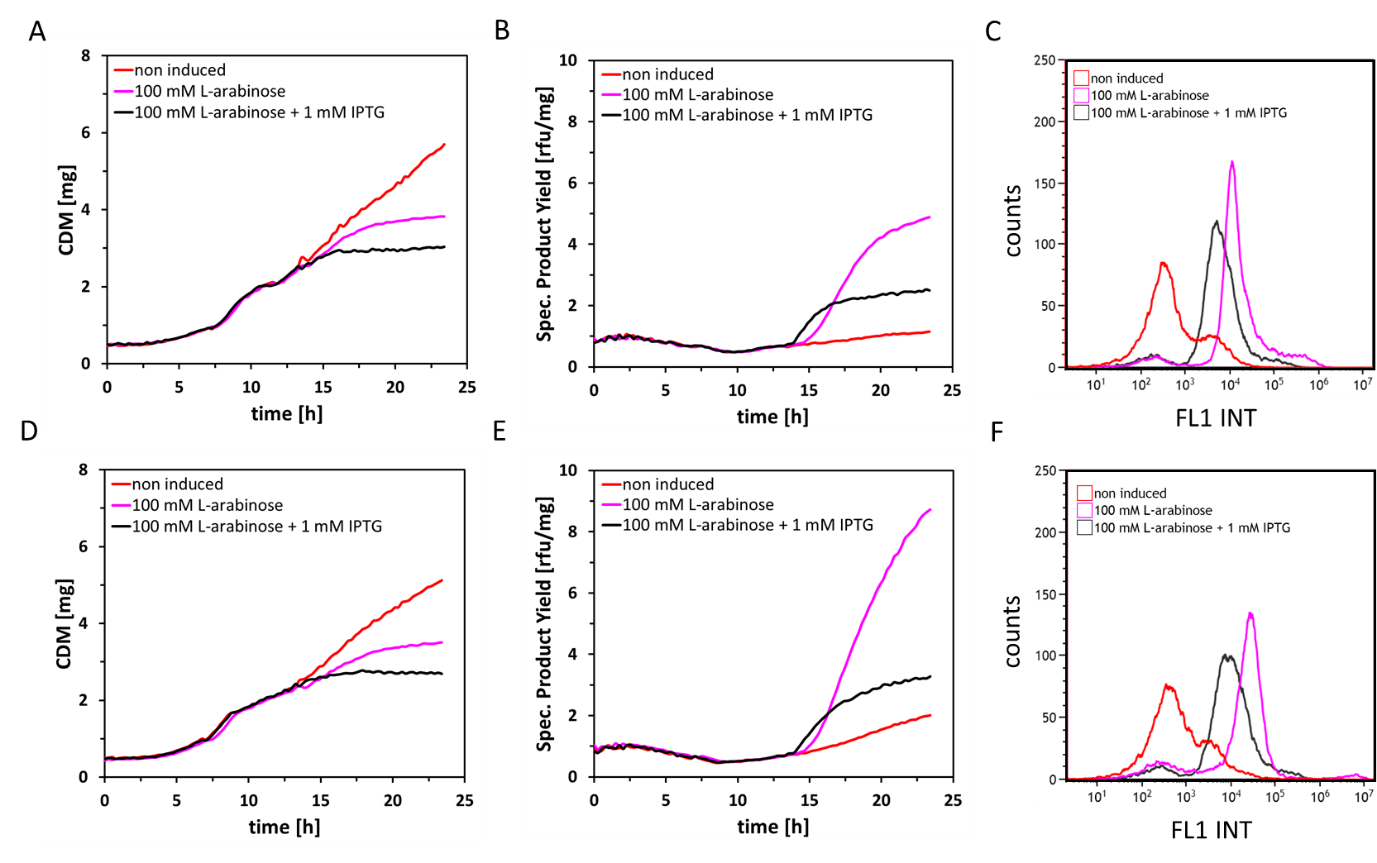


Supplementary Figure 16 Process characteristic showing product formation kinetics and flow cytometry analysis of E. coli strains BL21-AI (A, B, C) and BL21-AI<gp2> (D, E, F) expressing YliF-GFP fusion protein during fed-batch like cultivation. Induction was performed with 100 mM L-arabinose and 100 mM L-arabinose + 1 mM IPTG. The mean CDM [mg] and mean specific GFP yield [rfu/mg] represents duplicate samples. Flow cytometry results from a single experiment are presented.


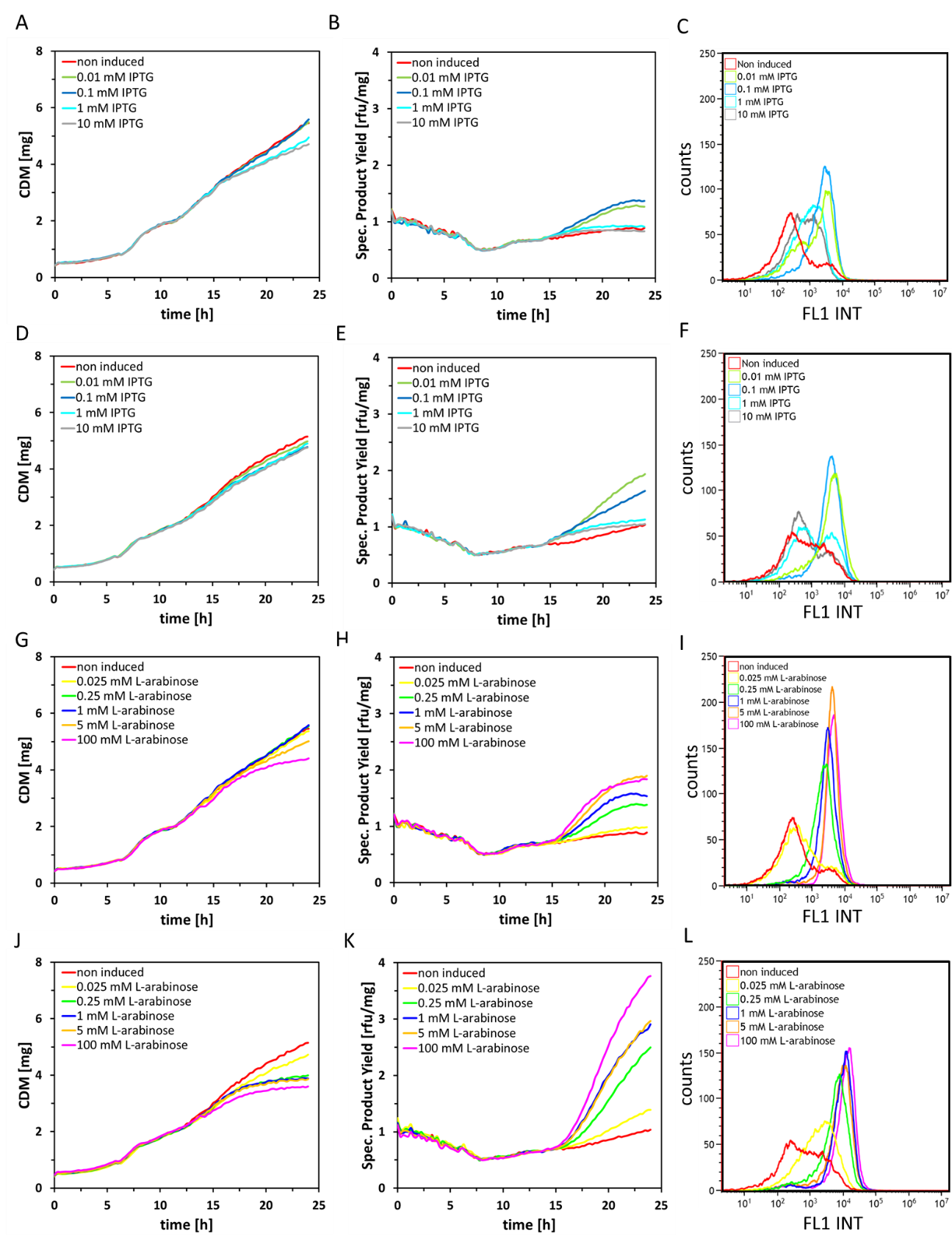


Supplementary Figure 17 Product formation kinetics and flow cytometry analysis of single‑cell expression of E. coli strains BL21-AI (**A, B, C, G, H, I**) and BL21-AI<gp2> (**D, E, F, J, K, L**) expressing YdiK-GFP fusion protein during fed-batch like cultivation. Induction was performed either with L-arabinose (0.025, 0.25, 1, 5, 100 mM) only (**G, H, I, J, K, L**) or with IPTG (0.01, 0.1, 1, 10 mM) only (**A, B, C, D, E, F**). The mean CDM [mg] and mean specific GFP yield [rfu/mg] represents duplicate samples. Flow cytometry results from a single experiment are presented.


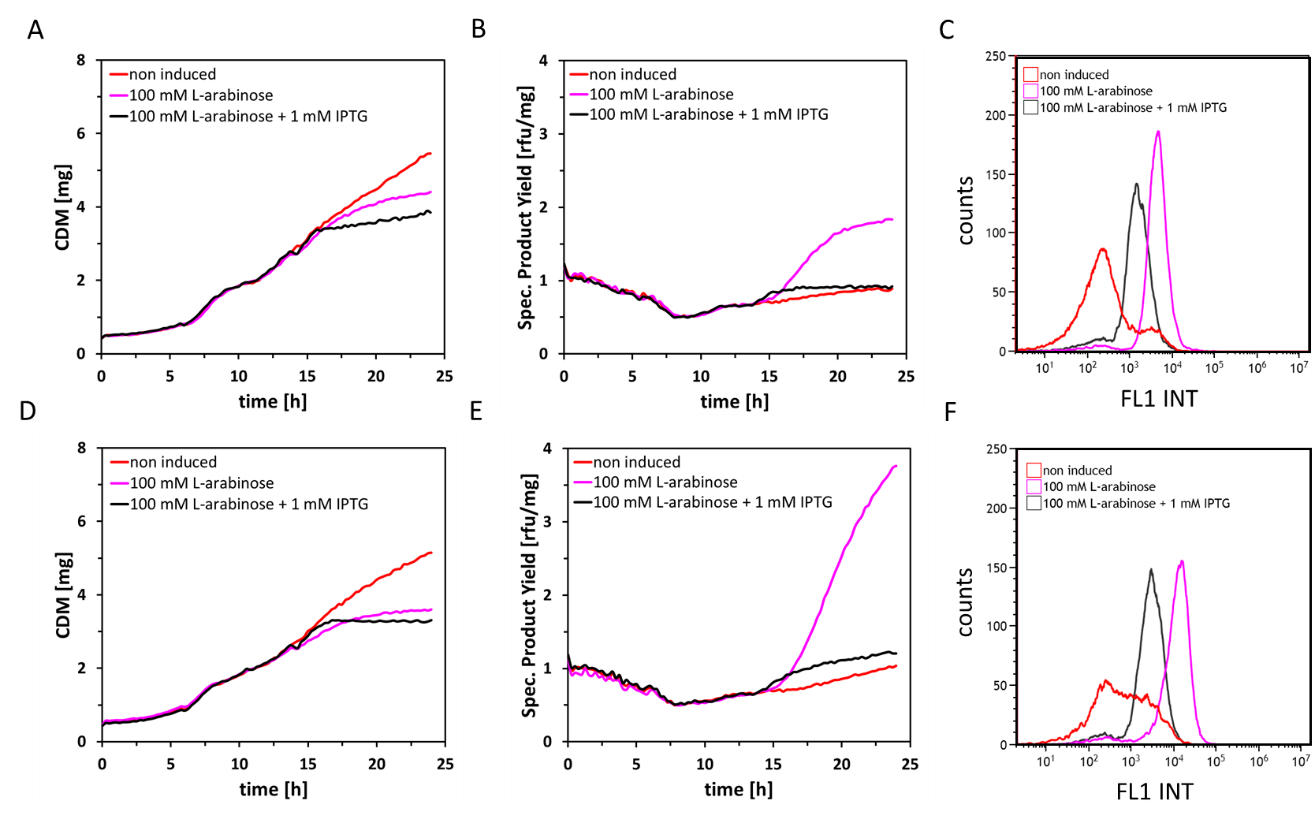


Supplementary Figure 18 Process characteristic showing product formation kinetics and flow cytometry analysis of E. coli strains BL21-AI (**A, B, C**) and BL21-AI<gp2> (**D, E, F**) expressing YdiK-GFP fusion protein during fed-batch like cultivation. Induction was performed with 100 mM L-arabinose and 100 mM L-arabinose + 1 mM IPTG. The mean CDM [mg] and mean specific GFP yield [rfu/mg] represents duplicate samples. Flow cytometry results from a single experiment are presented.


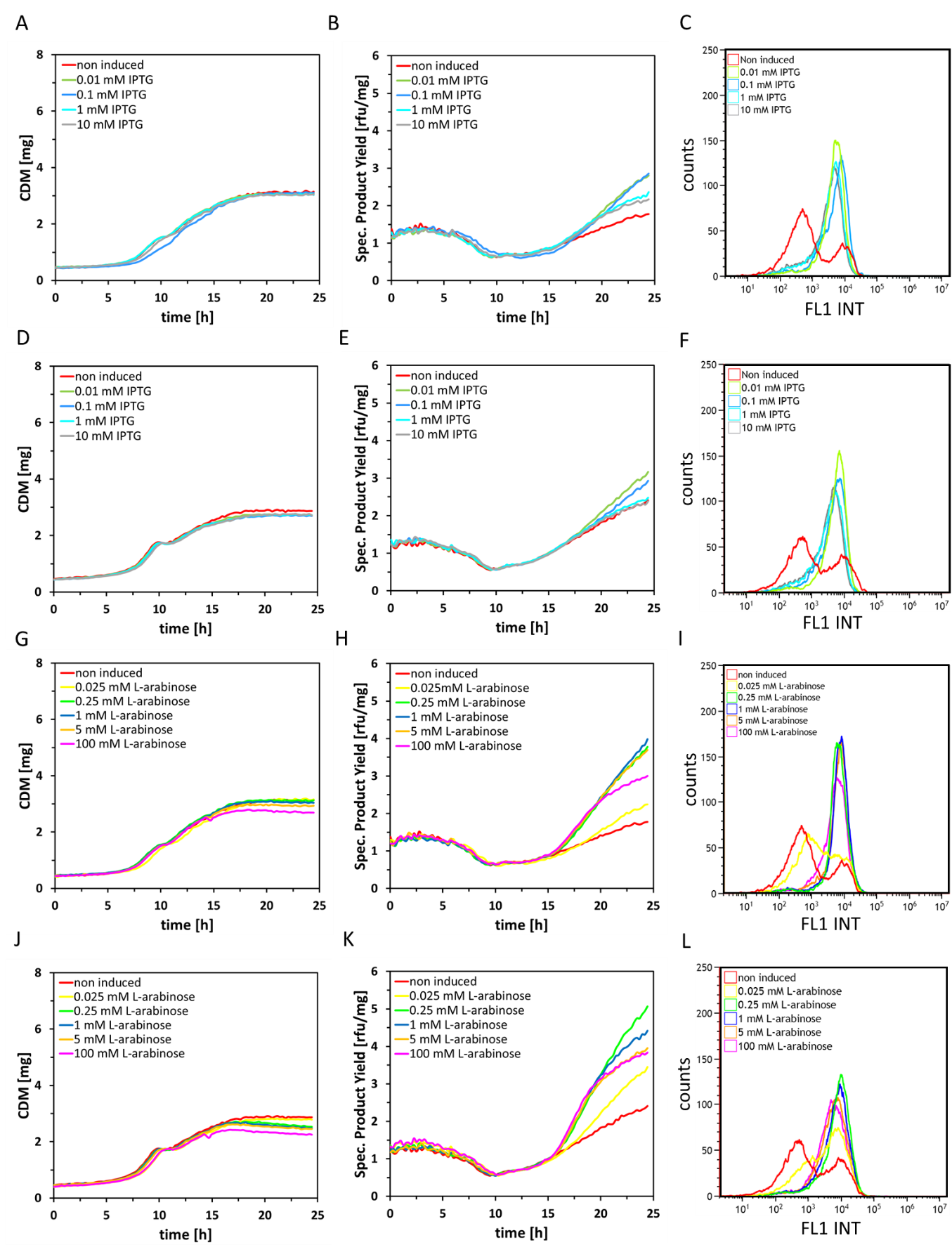


Supplementary Figure 19 Product formation kinetics and flow cytometry analysis of single‑cell expression of E. coli strains BL21-AI (**A, B, C, G, H, I**) and BL21-AI<gp2> (**D, E, F, J, K, L**) expressing YhhJ-GFP fusion protein during fed-batch like cultivation. Induction was performed either with L-arabinose (0.025, 0.25, 1, 5, 100 mM) only (**G, H, I, J, K, L**) or with IPTG (0.01, 0.1, 1, 10 mM) only (**A, B, C, D, E, F**). The mean CDM [mg] and mean specific GFP yield [rfu/mg] represents duplicate samples. Flow cytometry results from a single experiment are presented.


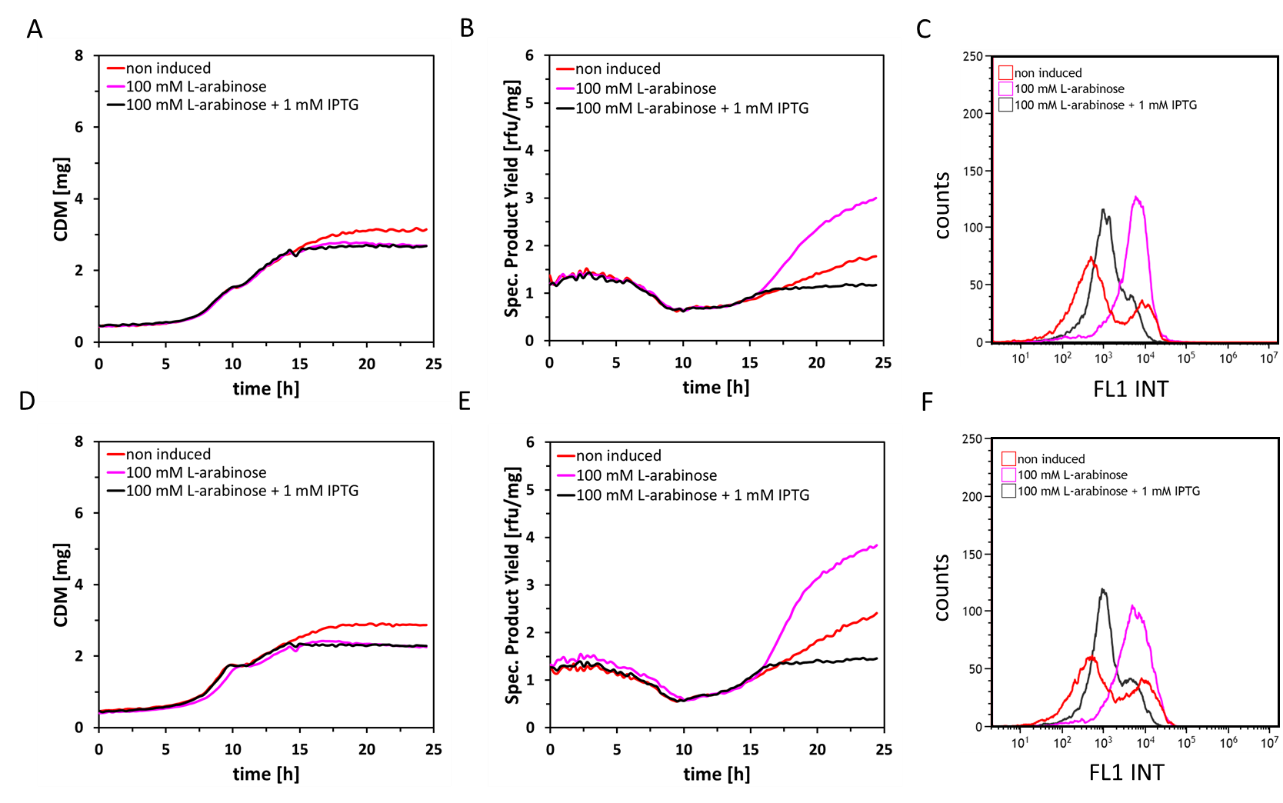


Supplementary Figure 20 Process characteristic showing product formation kinetics and flow cytometry analysis of E. coli strains BL21-AI (**A, B, C**) and BL21-AI<gp2> (**D, E, F**) expressing YhhJ-GFP fusion protein during fed-batch like cultivation. Induction was performed with 100 mM L-arabinose and 100 mM L-arabinose + 1 mM IPTG. The mean CDM [mg] and mean specific GFP yield [rfu/mg] represents duplicate samples. Flow cytometry results from a single experiment are presented.


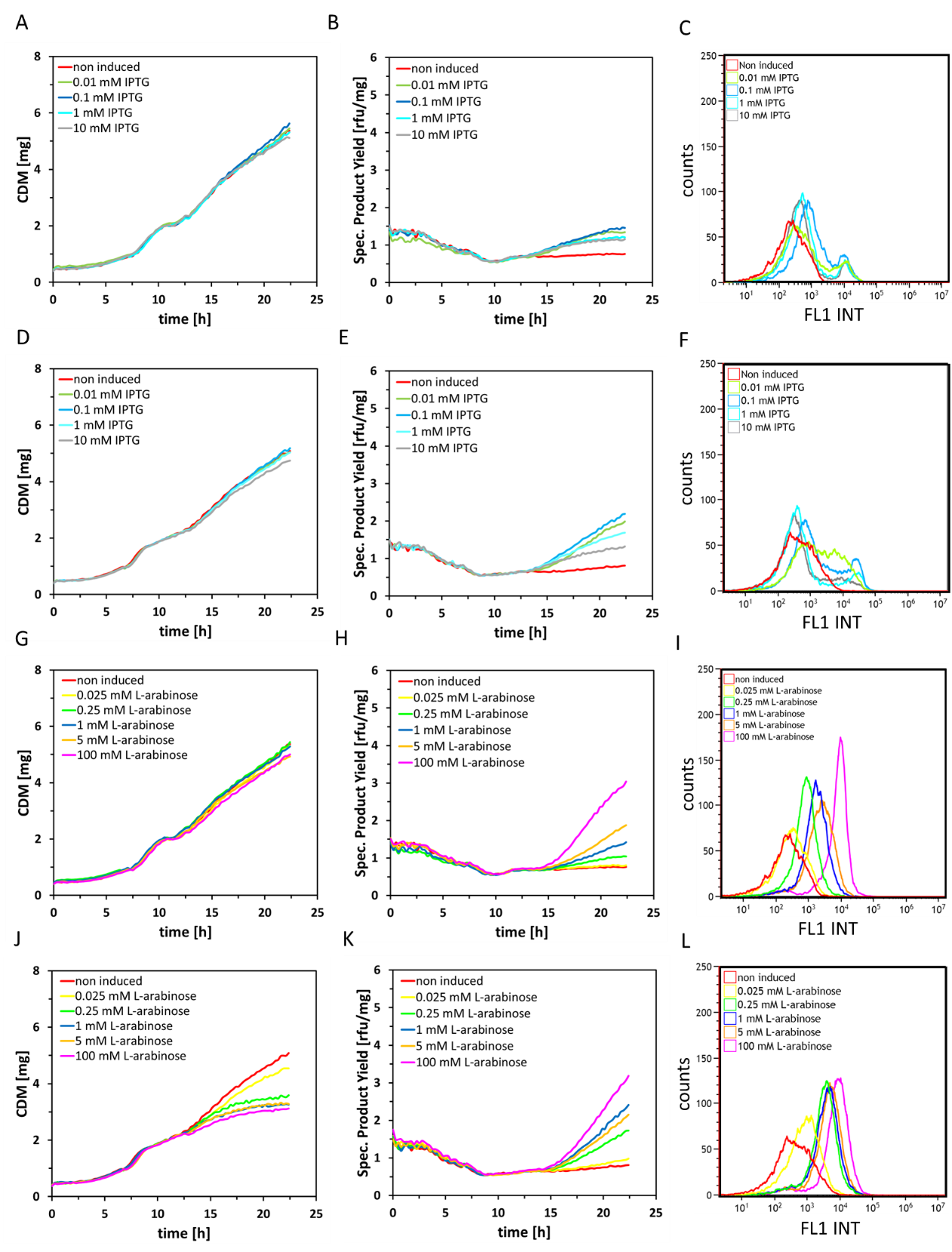


Supplementary Figure 21 Product formation kinetics and flow cytometry analysis of single‑cell expression of E. coli strains BL21-AI (**A, B, C, G, H, I**) and BL21-AI<gp2> (**D, E, F, J, K, L**) expressing YfbF-GFP fusion protein during fed-batch like cultivation. Induction was performed either with L-arabinose (0.025, 0.25, 1, 5, 100 mM) only (**G, H, I, J, K, L**) or with IPTG (0.01, 0.1, 1, 10 mM) only (**A, B, C, D, E, F**). The mean CDM [mg] and mean specific GFP yield [rfu/mg] represents duplicate samples. Flow cytometry results from a single experiment are presented.


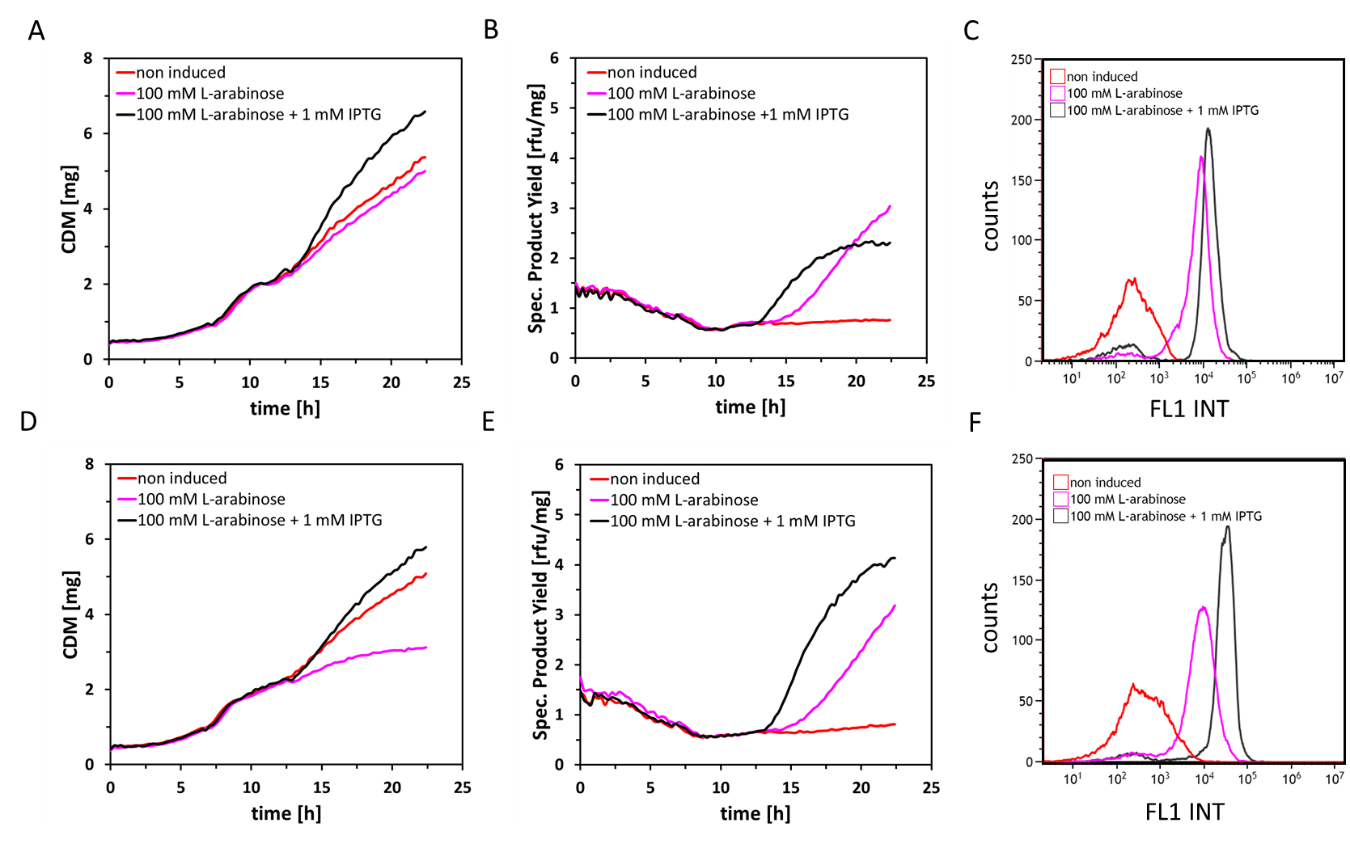


Supplementary Figure 22 Process characteristic showing product formation kinetics and flow cytometry analysis of E. coli strains BL21-AI (**A, B, C**) and BL21-AI<gp2> (**D, E, F**) expressing YfbF-GFP fusion protein during fed-batch like cultivation. Induction was performed with 100 mM L-arabinose and 100 mM L-arabinose + 1 mM IPTG. The mean CDM [mg] and mean specific GFP yield [rfu/mg] represents duplicate samples. Flow cytometry results from a single experiment are presented.

### Western Blot (detection with anit GFP antibody)

#### YhdY-GFP

Uniprot: P45768

Inner membrane amino-acid ABC transporter permease protein YhdY


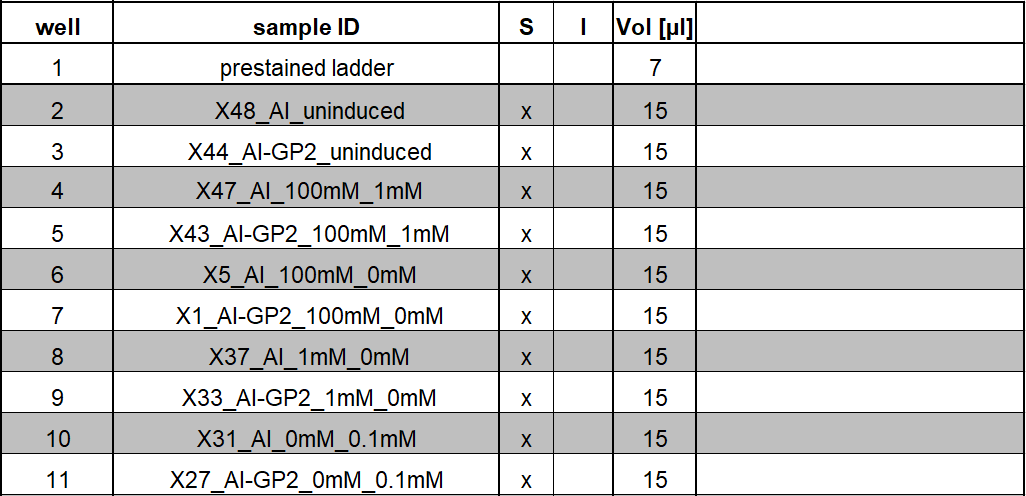


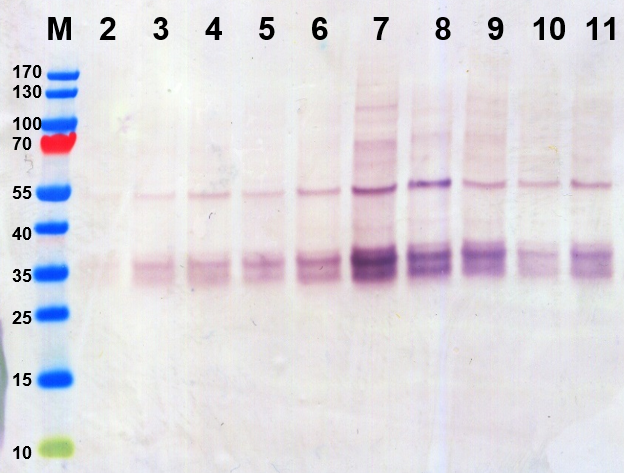


Supplementary Figure 23 Western Blot showing overexpression of YhdY-GFP during fed-batch cultivations of E. coli strain BL21-AI and BL21-AI<gp2>. GFP capturing was performed with Anti-GFP antibody and detected with alkaline phosphatase‐labeled anti‐mouse IgG.

#### Psta-GFP

Uniprot: P07654

Phosphate transport system permease protein PstA

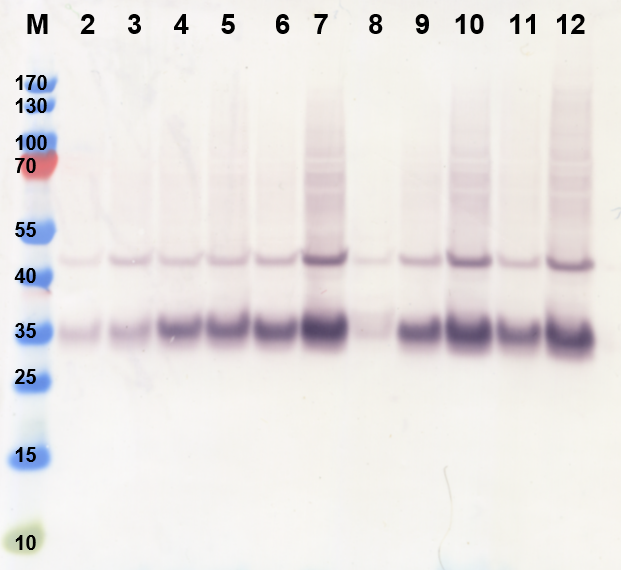

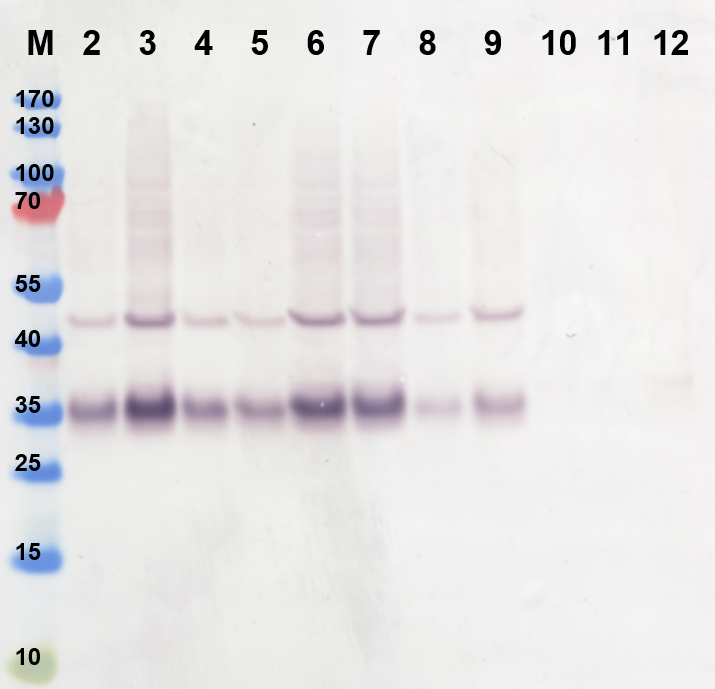


Supplementary Figure 24 Western Blot showing overexpression of PstA-GFP during fed-batch cultivations of E. coli strain BL21-AI and BL21-AI<gp2>. GFP capturing was performed with Anti-GFP antibody and detected with alkaline phosphatase‐labeled anti‐mouse IgG.

#### YliF-GFP

Uniprot: P75801

Probable diguanylate cyclase DgcI


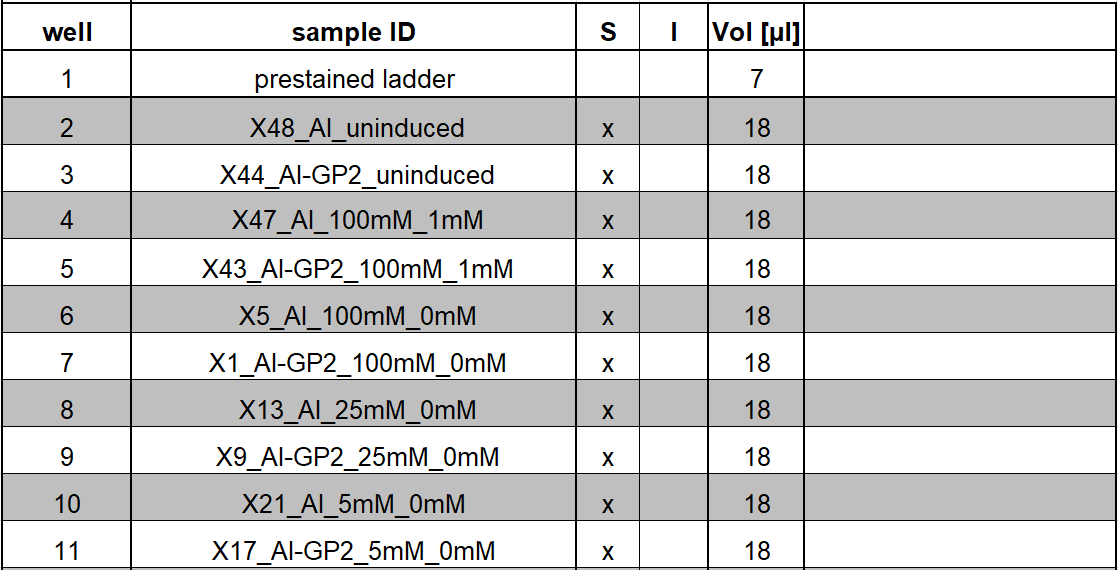

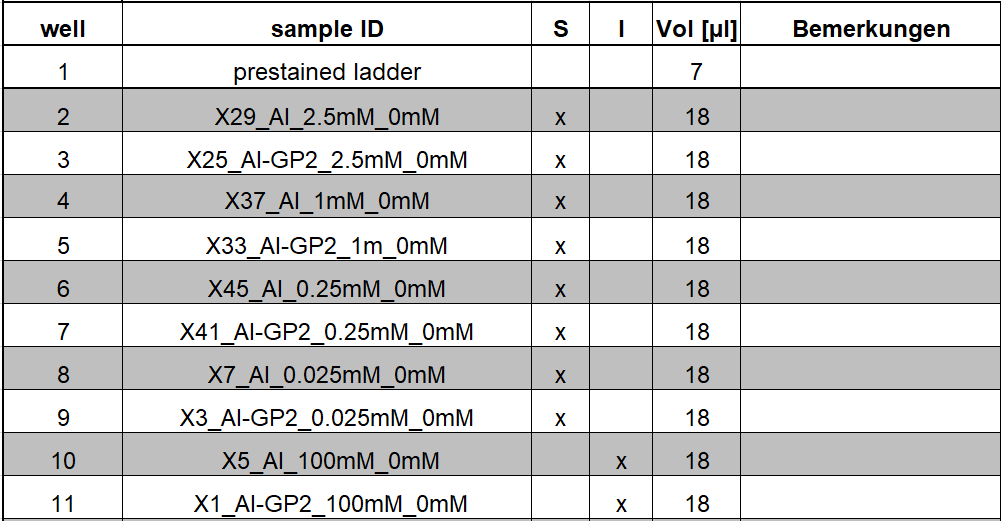


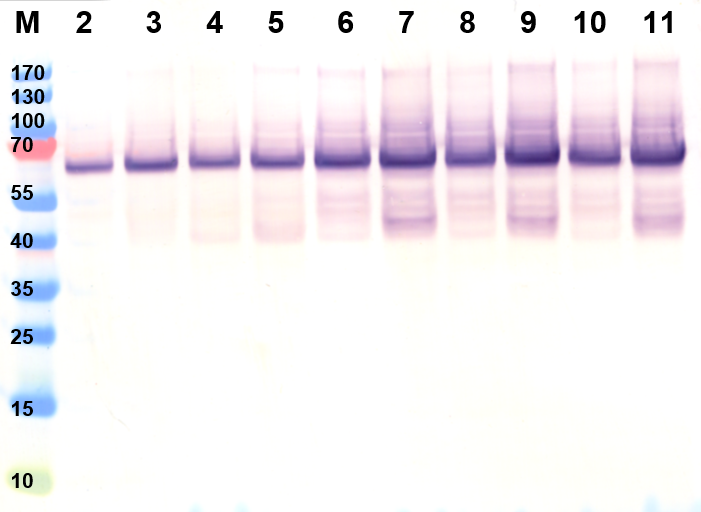

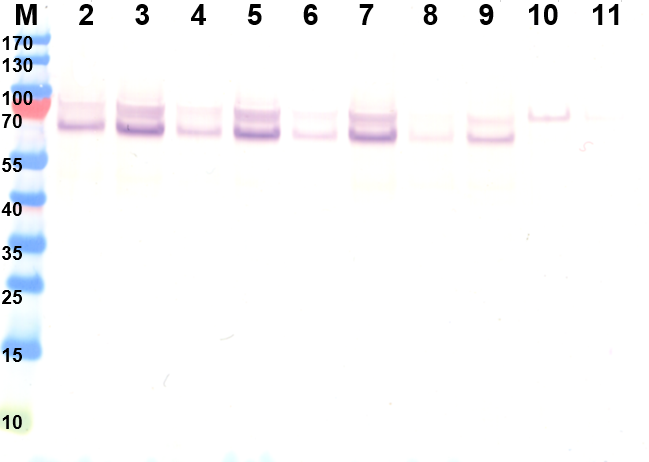


Supplementary Figure 25 Western Blot showing overexpression of YliF-GFP during fed-batch cultivations of E. coli strain BL21-AI and BL21-AI<gp2>. GFP capturing was performed with Anti-GFP antibody and detected with alkaline phosphatase‐labeled anti‐mouse IgG.

#### YdiK-GFP

Uniprot: P0AFS7

Putative transport proteine YdiK


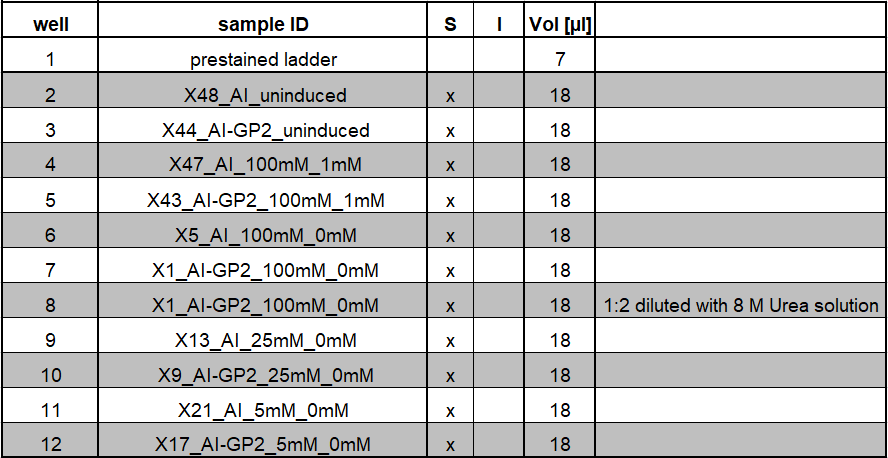

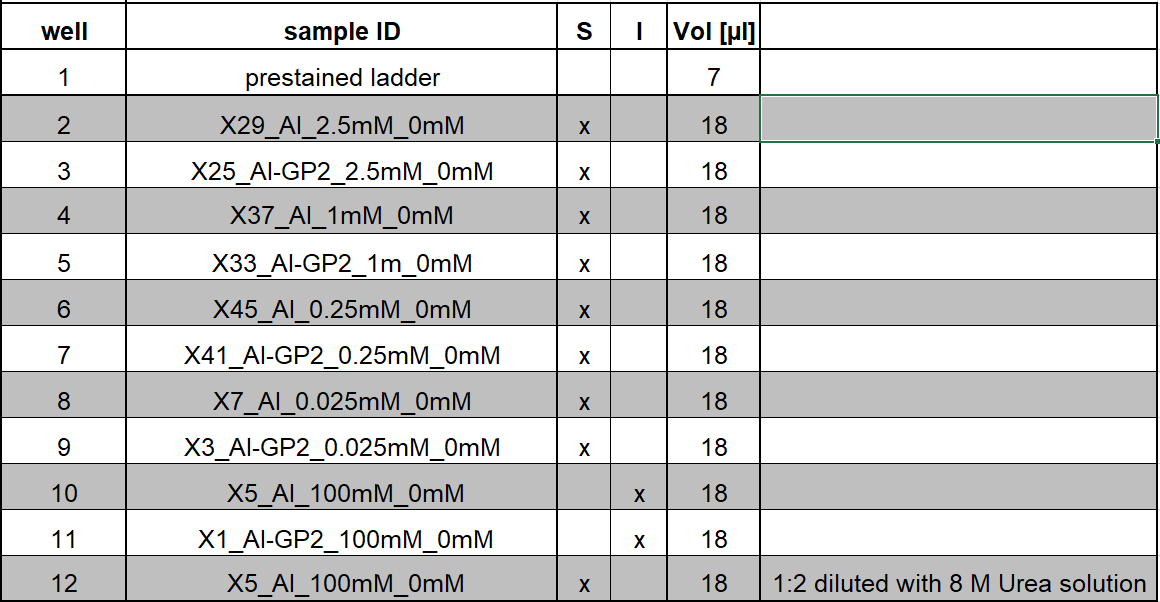


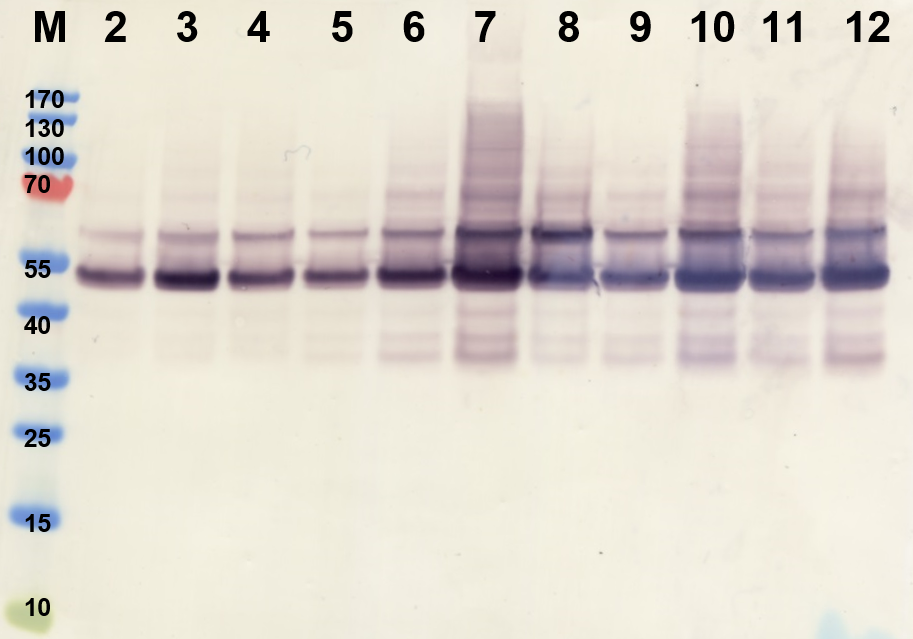

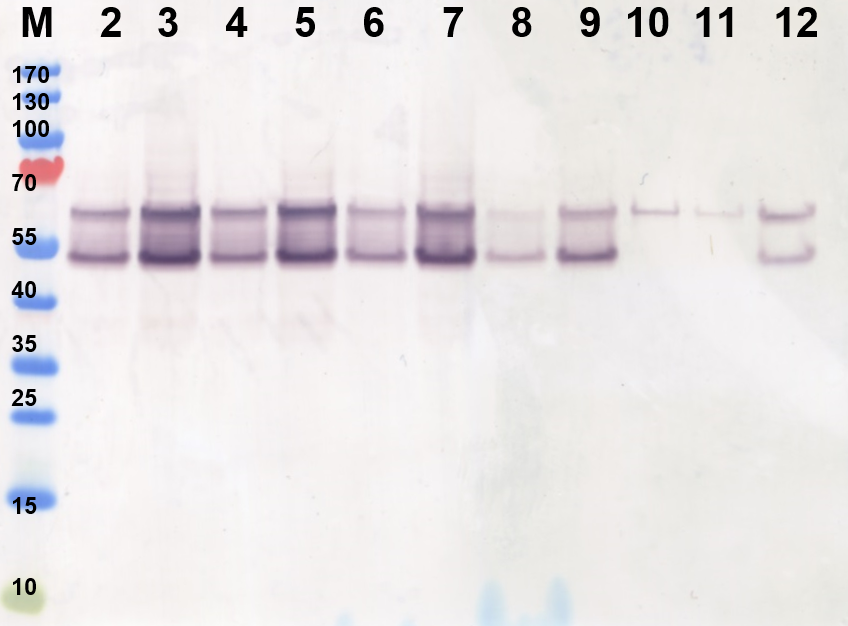


Supplementary Figure 26 Western Blot showing overexpression of YdiK-GFP during fed-batch cultivations of E. coli strain BL21-AI and BL21-AI<gp2>. GFP capturing was performed with Anti-GFP antibody and detected with alkaline phosphatase‐labeled anti‐mouse IgG.

#### YfbF-GFP

Uniprot: P77757

Undecaprenyl-phosphate 4-deoxy-4-formamido-L-arabinose transferase


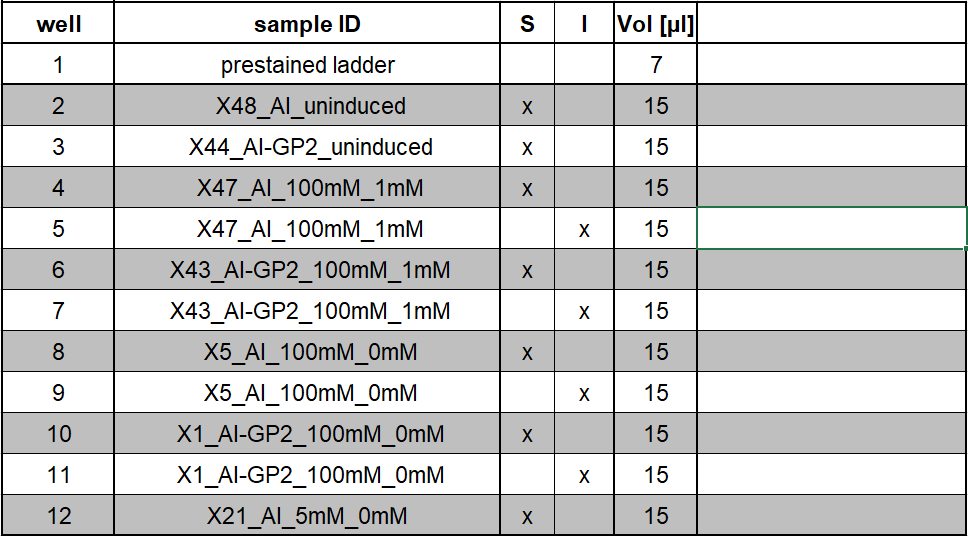

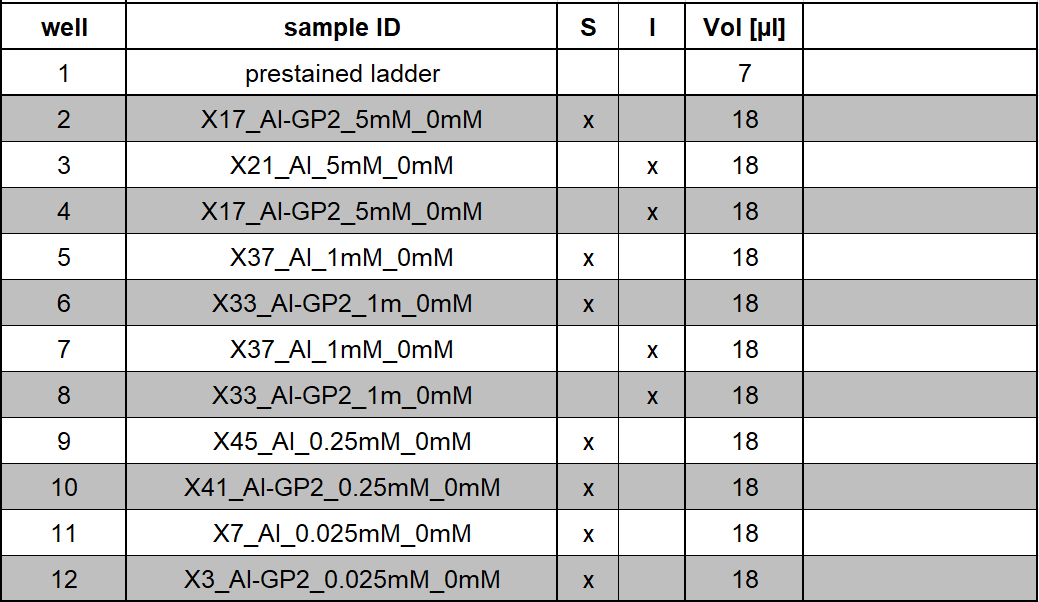


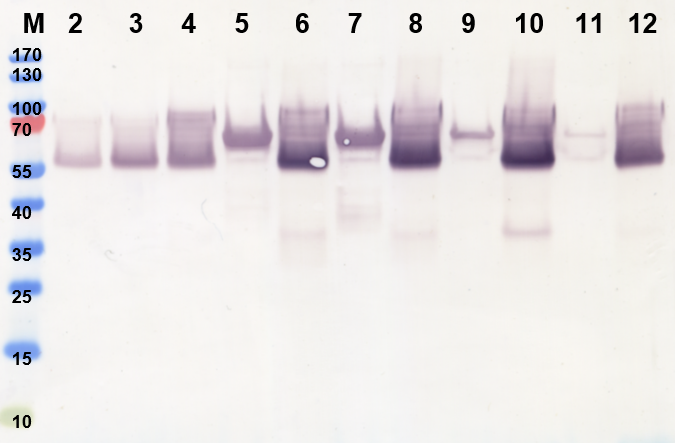

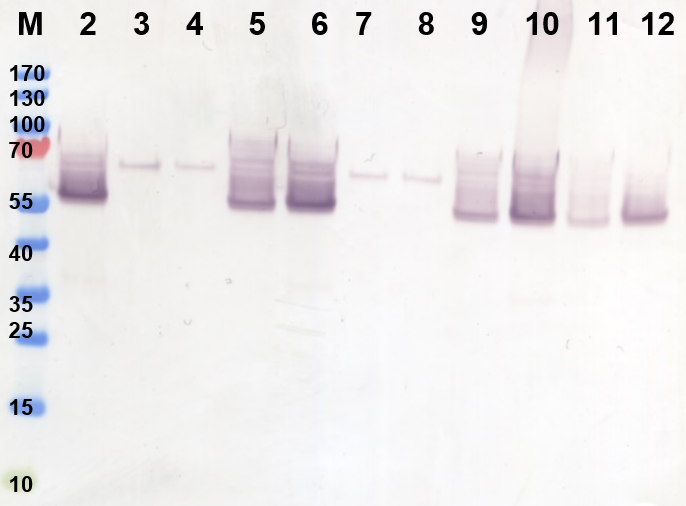


Supplementary Figure 27 Western Blot showing overexpression of YfbF-GFP during fed-batch cultivations of E. coli strain BL21-AI and BL21-AI<gp2>. GFP capturing was performed with Anti-GFP antibody and detected with alkaline phosphatase‐labeled anti‐mouse IgG.

#### YhhJ-GFP

Uniprot: P0AGH1

Inner membrane transport permease YhhJ


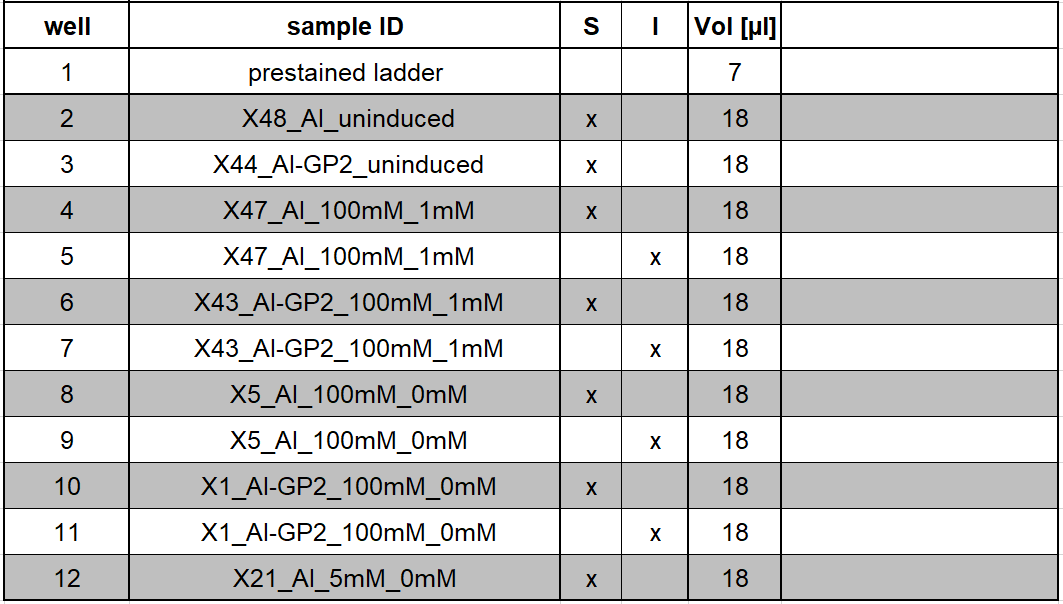

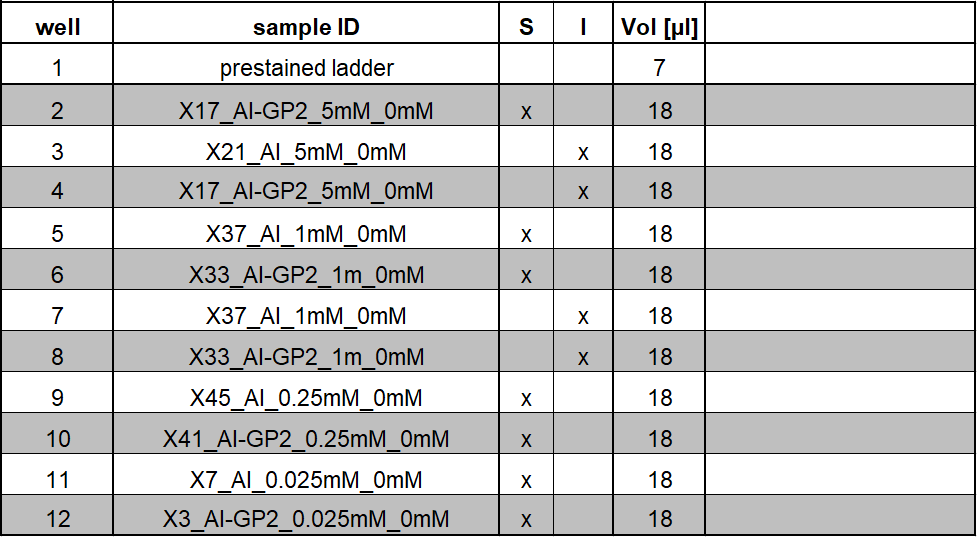


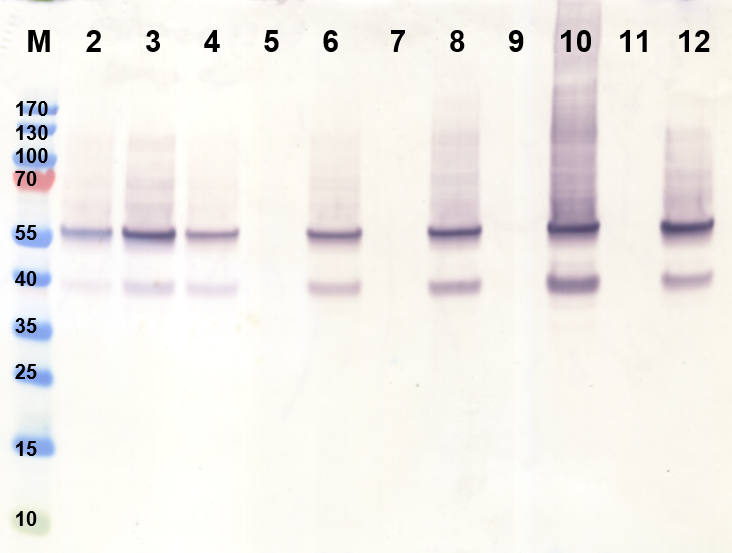

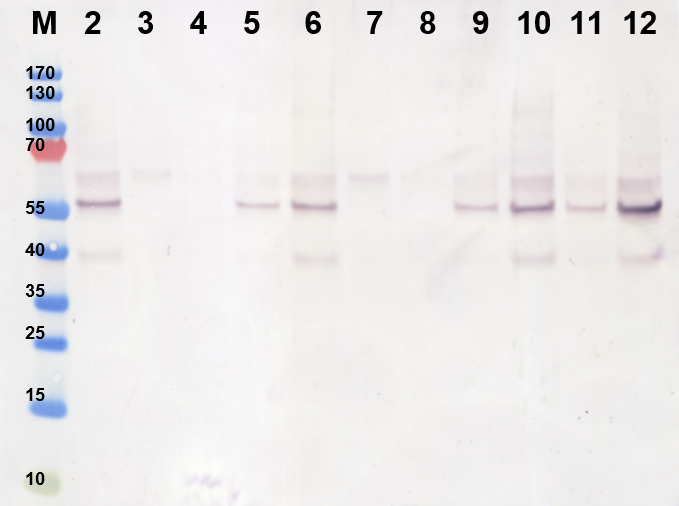


Supplementary Figure 28 Western Blot showing overexpression of YhhJ-GFP during fed-batch cultivations of E. coli strain BL21-AI and BL21-AI<gp2>. GFP capturing was performed with Anti-GFP antibody and detected with alkaline phosphatase‐labeled anti‐mouse IgG.
